# An *m*/*z*- and Intensity-Based HRMS Clustering Algorithm Targeted toward Single-Cell Metabolomics

**DOI:** 10.1021/jasms.6c00150

**Published:** 2026-07-10

**Authors:** Dirk Wevers, Laura Koldenhof, Laura Castaneda, Anouschka van der Zeeuw, Hellen Fass, Thomas Hankemeier, Charlie Clark, Ahmed Ali

**Affiliations:** † Metabolomics and Analytics Centre, Leiden Academic Centre for Drug Research, Wassenaarseweg 76, AL Leiden 2333, The Netherlands; ‡ 4496 Universiteit Utrecht, Heidelberglaan 8, CS Utrecht 3584, The Netherlands; § Hogeschool Leiden, Zernikedreef 11, CK Leiden 2333, The Netherlands

**Keywords:** single-cell metabolomics, HRMS data processing, reference-free clustering and alignment, noise filtering, peak splitting, aggregation

## Abstract

Single-cell (SC) metabolomics holds great potential in the development of novel diagnostic tools and mechanistic insights into cell biology. Using high-resolution mass spectrometry (HRMS), the masses of a single cell’s constituents can be determined with an accuracy high enough to derive their respective elemental compositions. Using a molecule’s mass and its MS fragmentation pattern, in many cases a molecular structure can be assigned or looked up in databases. Due to the small measurement cell volume of an Orbitrap HRMS instrument, samples need to be scanned multiple times, which necessitates across-scan clustering per sample, and across-sample alignment of *m*/*z* values. However, existing HRMS data processing software is not designed to process SC HRMS data, as it typically requires liquid chromatography retention times or reference spectra for *m*/*z* clustering and alignment. Herein, a novel, robust SC HRMS *m*/*z* clustering and alignment algorithm is presented and compared with two commercially available and industry standard algorithms used by Sciex MarkerView and Thermo FreeStyle. Furthermore, output is compared with clustering results from DBSCAN and MaldiQuant binning. Our algorithm, Global Clustering unTargeted Analysis (GCTA), enforces a strict maximum on the cluster size, thereby reducing the chance of peak aggregation. Furthermore, by design, GCTA enables noise filtering based on intensity and number of peaks. Across-scan clustering and across-sample alignment were contrasted for accuracy in finding peaks identified by commercial software output and peaks with known *m*/*z* values corresponding to standards and HMDB and LipidMaps database hits. Comparisons are made based on data recorded for quality control samples containing standard mixes as well as SC HRMS data recorded for two different cell lines. This work shows that the presented algorithm is comparable in accuracy with respect to MarkerView and FreeStyle, reliably identifies compounds, is less prone to peak splitting than MaldiQuant binning while providing similar levels of error in clustering peaks, and successfully filters noise. Furthermore, it is shown to be competitive with DBSCAN, MaldiQuant binning and MarkerView when compared to theoretical *m*/*z* values based on database hits. GCTA encompasses both *m*/*z* clustering and reference-free alignment, which makes it pivotal to further development of untargeted SC HRMS metabolomics.

## Graphical abstract

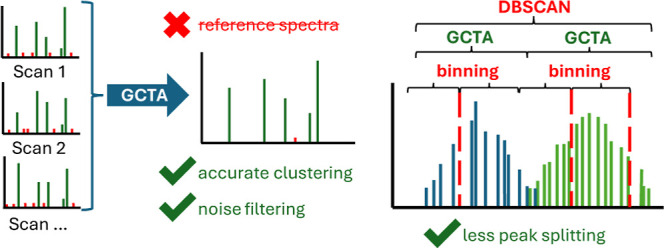

## Introduction

Cells are the fundamental units of life, and each cell is unique, even when their genetic information is the same.^1^ This single-cell (SC) heterogeneity has implications for disease subtype^2,3^ and consequently, treatment responsivity.^4^ Therefore, there is a need for analytical platforms that are capable of the analysis of single cells. To get the most accurate depiction of the cellular phenotype, SC metabolomics is often used.^5^ SC metabolomics aims to investigate the metabolome, which consists of all small molecules that are converted or produced in the chemical reactions occurring in a cell. However, SC metabolomic analysis is complicated due to low analyte concentrations, small sample volumes and large structural and chemical diversity of metabolites in single cells.^6^ Due to its high sensitivity and accuracy, high-resolution mass spectrometry (HRMS) is the analytical platform of choice in SC metabolomics. Despite this, SC metabolomics by HRMS still suffers from its own set of unique challenges, especially in the processing and analysis of SC HRMS data sets.^7^

In SC metabolomics, live adherent cells are typically sampled under a microscope using a micromanipulator and a platinum-coated glass capillary. The collected cells are then analyzed using direct-infusion HRMS, most often on Orbitrap instruments. Due to the high resolution of these systems, the same compound can appear with slightly different mass-to-charge ratio (*m*/*z*) values across individual scans. To make the data useable, *m*/*z* values need to be clustered across scans so each compound is represented by a single *m*/*z* value. This issue exists across samples as well, where slightly different *m*/*z* values can be recorded for the same compound in different cells. Therefore, alignment across samples is also necessary. Both scan-to-scan and sample-to-sample *m*/*z* variability must be addressed to enable meaningful downstream analysis, such as identifying compounds that distinguish between biological conditions or sample groups. Alignment and clustering differ in that, in alignment, data points are moved along one or multiple data axes, whereas multiple data points are combined into a single data point in clustering. When alignment and clustering are not done (properly), unequal *m*/*z* values that correspond to the same compound are considered different features, thereby leading to a vast expansion of the data matrix and making downstream feature identification impossible.

A wide variety of data processing tools for HRMS data in metabolomics such as METASPACE^8^ are available; however, due to the noisy nature of SC metabolomics data, these tools are not recommended for use in all cases. Feature detection algorithms developed for LC–MS data such as GridMass^9^ and region of interest searching^10^ can be unsuitable as they typically require retention time data and some sort of relation between consecutive spectra, which is not necessarily true for SC HRMS data. Furthermore, current clustering methods rely on some sort of binning,^11^ i.e. defining edges on a given data axis between which data points (from different scans of the same sample, for instance) are grouped into a single data point. Binning quality critically relies on the choice of bin edges: when a peak is located around the edge of a bin, this can lead to peak splitting. Conversely, data points that correspond to different peaks may be combined into a single peak when they are located between two bin edges, which would mean information can be lost and consequently, mass accuracy drops, complicating downstream feature identification. Alternatively, density-based clustering methods such as DBSCAN^12,13^ and OPTICS^14^ look along an axis for regions that are information-dense. The major difference between binning and density-based clustering is that binning relies on the use of equidistant edges between which data points are grouped together, whereas density-based approaches define the location of a peak based on the density of the data points along the axis, i.e. high-density regions are indicative of the presence of a peak. Typically, these algorithms rely on the user to define a search radius and a maximum number of data points to be found within that region. The choice of these values strongly influences the results, which hampers the application in untargeted experiments. Furthermore, application of for instance DBSCAN is hampered by density-connectivity, which is the aggregation of clusters into large clusters if they share one or more data points; this is also clarified in Figure S15. This density-connectivity leads to a loss of information as peaks are combined into a single peak (compound). This also complicates identification, as the combined *m*/*z* value may not be present in a database (or is by coincidence). Typically, these clustering algorithms do not consider the intensity corresponding to the different *m*/*z* values in *m*/*z* clustering. After clustering per file, alignment^15–17^ across files is needed; however, most available alignment algorithms require reference spectra, which are not available in SC metabolomics studies as the sample is typically consumed entirely during analysis. Furthermore, it is not possible to generate a pooled sample before analysis, due to the limited sample volume available. Lastly, clustering algorithms like Thermo FreeStyle and Sciex’s MarkerView are proprietary, and therefore their inner workings remain unknown, whereas the clustering and alignment introduced herein is publicly available via GitHub (see Data and Code Availability Statement).

Herein, a novel clustering and alignment algorithm *Global Clustering unTargeted Analysis* (GCTA) is presented and applied to SC metabolomics data recorded on a ThermoFischer Orbitrap Exploris 480 instrument. GCTA is a density-based clustering algorithm that enforces a strict maximum size *K* per cluster, thereby preventing the aggregation of clusters corresponding to multiple compounds. The pseudocode for this algorithm is given in *Code snippet* S1, the inner workings of the algorithm are also illustrated in Figure 1. For each data point in a scan, a user-defined maximum number of neighboring points *K* within a certain distance *eps* are grouped into a neighborhood; in this way, the probability of incorrect splitting or grouping is reduced. Each neighborhood is assigned a score calculated based on the number of neighbors and the intensity of the mean *m*/*z* value. Functions are provided for score calculation in both across-scans and across-files filtering, and users can design functions themselves as well. Subsequently, based on their scores, all neighborhoods are looped through, and the neighborhoods with the highest scores are clustered into peaks. Proper setting of the score threshold can also be a means of noise filtering, enabling GCTA to integrate noise filtering and data clustering. A systematic way to choose the score threshold is visualized in Figure 8, i.e. by comparing the intensities of the neighborhoods that are removed and retained for different score thresholds. The code needed for this endeavor can be found in the aforementioned GitHub. Peaks that appeared less than a certain number of times across scans can be tagged for potential removal. After clustering across scans, GCTA also aligns *m*/*z* values across files. Lastly, it makes use of the (clustered) intensities of the different *m*/*z* values in their clustering. To the best of our knowledge, this seamless integration of clustering and noise filtering sets GCTA apart from other clustering or noise filtering algorithms.

**Figure 1:**
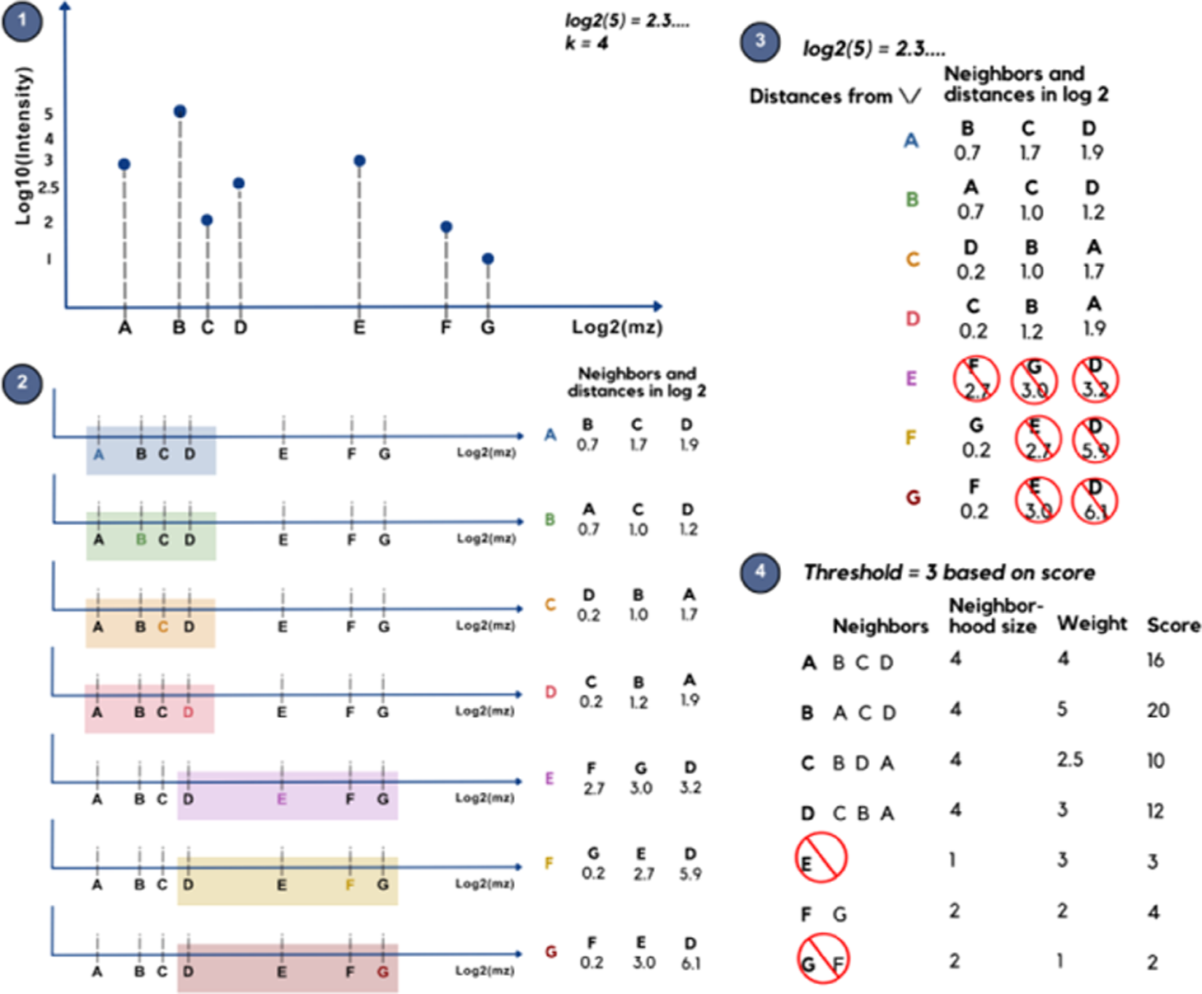
Description of the code of the GCTA algorithm. (1) Raw scan spectrum (2) For each point, the distances on a log-scale to all other data points are calculated in ppm; based on these mutual distances, a user-defined number of points (herein: *k* = 4) are grouped into neighborhoods. (3) Data points further away than *log*_2_ *x* ppm are excluded; neighborhood sizes can be determined. (4) Scores are calculated as the product of neighborhood size and weight, the weight being the *log*_10_ *I* of the reference *m*/*z* data point; neighborhoods with a score below the user-defined threshold (herein: 3) are excluded. Clusters are formed based on these scores in ascending order; points can be reassigned to a different cluster in this process.

**Figure 2:**
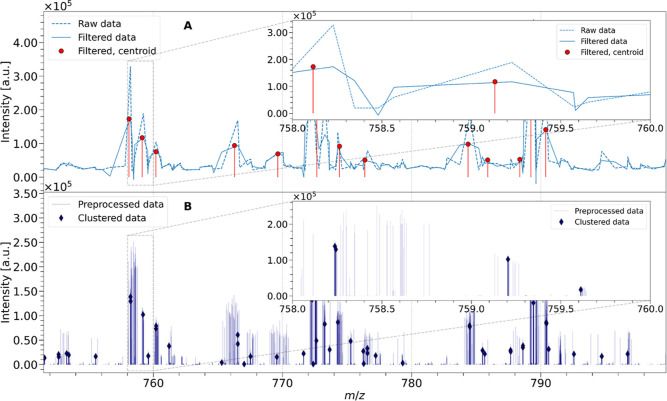
Data preprocessing workflow. (A) Raw data is smoothened using a Savitzky–Golay filter (parameter values in section Data generation and preprocessing) and centroided. (B) Subsequently, preprocessed data is clustered across scans using GCTAn; data from the individual scans is made transparent to show clearly which regions are more data-dense.

**Figure 3:**
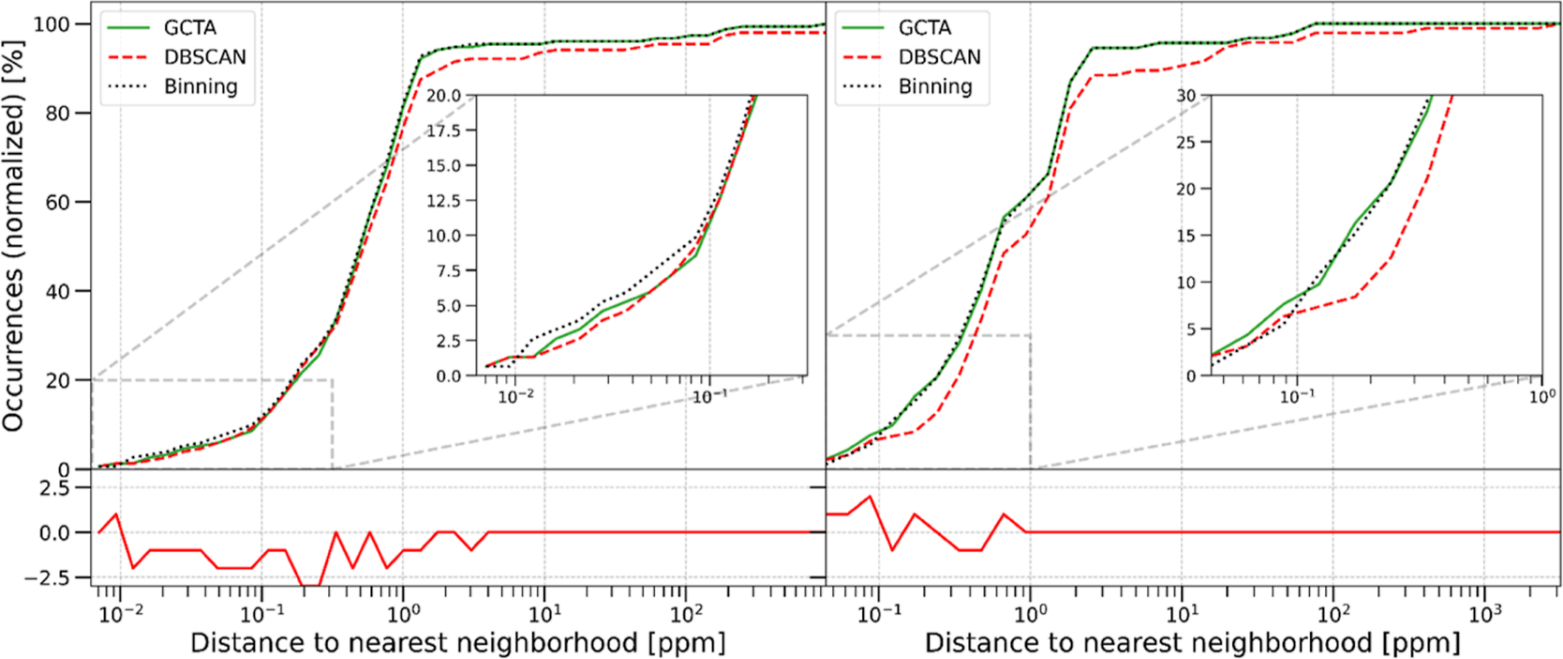
Clustering errors for GCTA, binning and DBSCAN for standards in QC files in positive (left) and negative mode. Data points represent normalized heights of cumulative histogram bars generated based on the resulting errors [ppm] after across-scan clustering; bin widths have been calculated using the Freedman-Diaconis rule (eq 1). Curves reaching higher normalized occurrences earlier (i.e., for lower distances) indicates more accurate algorithm performance. Red curve shows the difference GCTA [minus] binning. Note the logarithmic *x*-axis.

**Figure 4:**
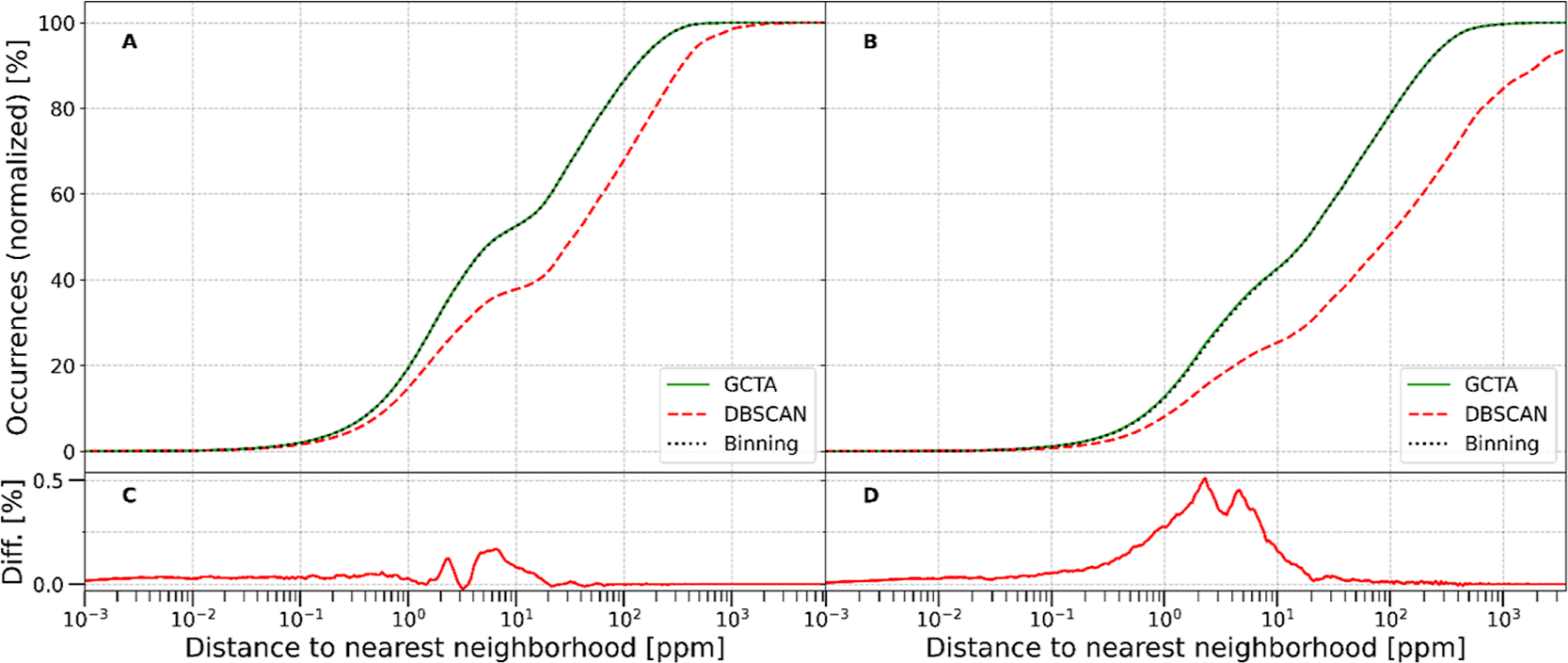
Clustering errors for GCTA, binning and DBSCAN relative to Thermo-extracted*m*/*z*values in cellular MS data files in (A,C) positive and (B,D) negative mode. Data points represent normalized heights of cumulative histogram bars generated based on the resulting errors [ppm] after across-scan clustering; bin widths have been calculated using the Freedman-Diaconis rule. Curves reaching higher normalized occurrences earlier indicates lower clustering error and hence better algorithm performance. Figures C and D show the difference GCTA [minus] binning for (C) positive and (D) negative-mode data. Note the logarithmic *x*-axis.

**Figure 5:**
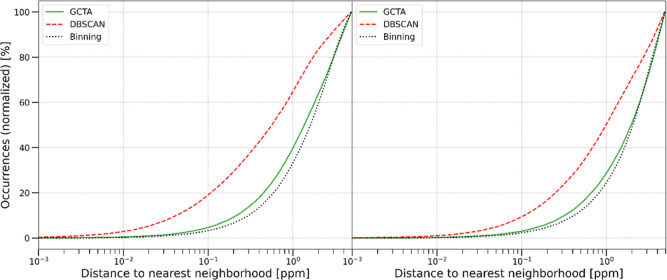
Alignment errors for GCTA, binning and DBSCAN relative to MarkerView-extracted *m*/*z* values in cellular MS data files in (left) positive and negative mode. Errors after across-file alignment are shown and only errors ≤5 ppm are considered. Data points represent normalized heights of cumulative histogram bars generated based on the resulting errors [ppm] after across-file clustering; bin widths have been calculated using the Freedman-Diaconis rule. Curves reaching higher normalized occurrences earlier indicates lower clustering error and hence better algorithm performance. Note the logarithmic *x*-axis.

**Figure 6:**
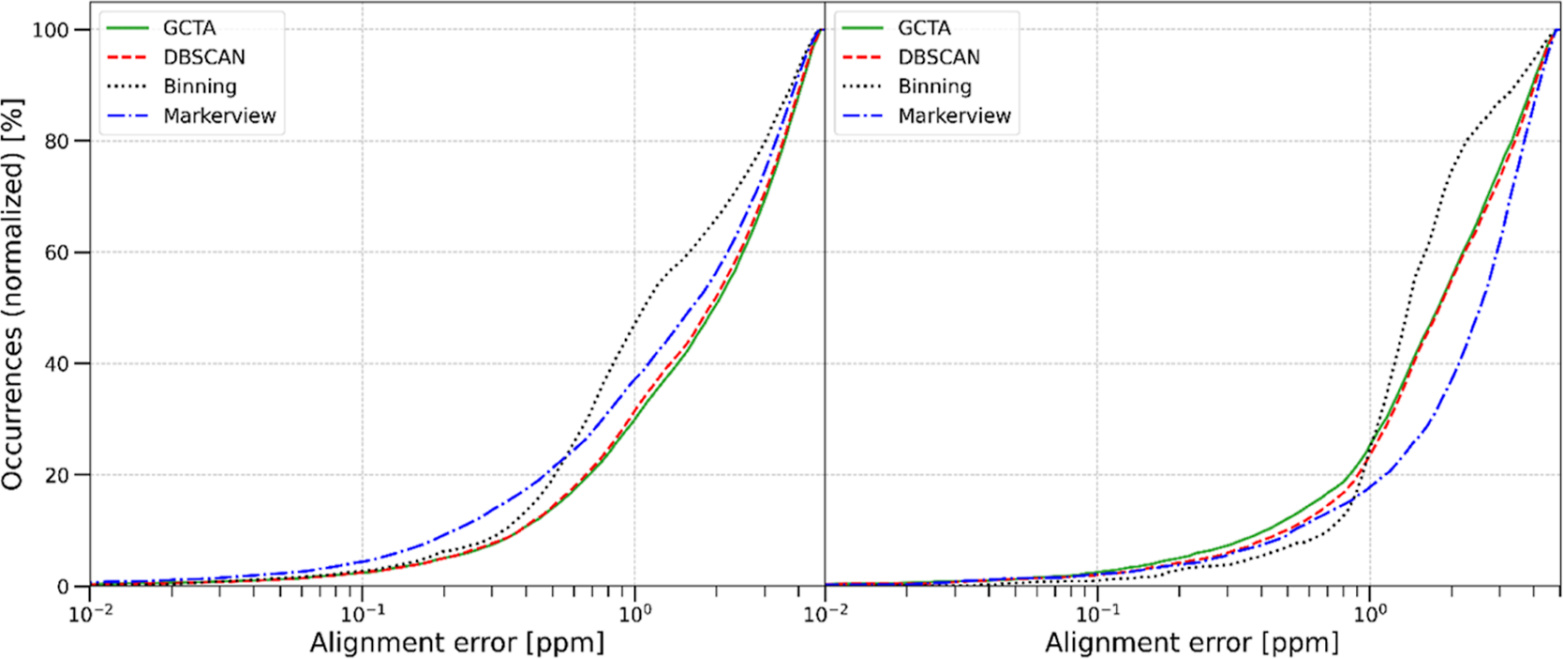
Alignment errors with respect to nearest HMDB hits for GCTA, binning, DBSCAN and MarkerView for (left) positive- and (right) negative-mode SCMS data. Only *m*/*z* values with an error ≤5 ppm with respect to the nearest database hit are considered. Data points represent normalized heights of cumulative histogram bars generated based on the resulting errors [ppm] after across-scan clustering; bin widths have been calculated using the Freedman-Diaconis rule. Curves reaching higher normalized occurrences earlier indicates lower clustering error and hence better algorithm performance. Note the logarithmic *x*-axis.

**Figure 7:**
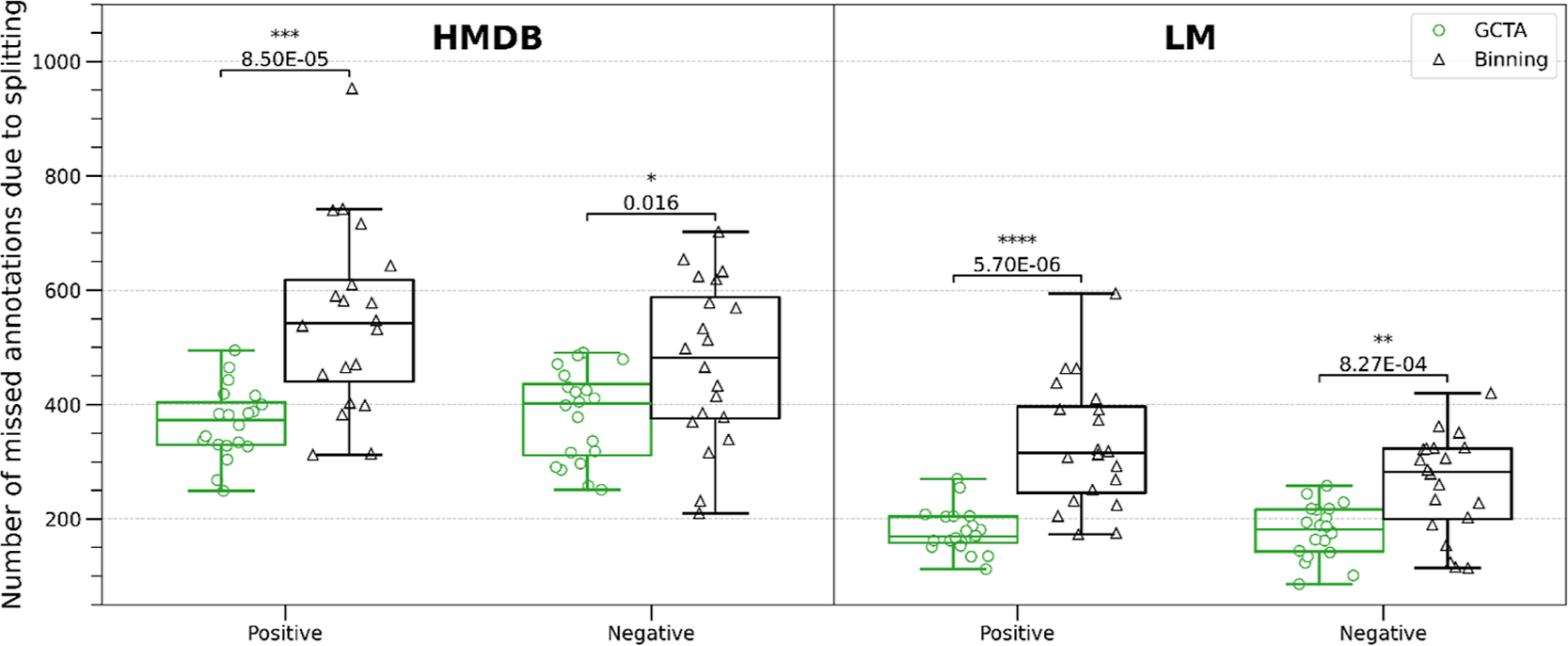
Numbers of missed annotations due to peak splitting by GCTA or MaldiQuant binning. Numbers of missed annotations are given per SC HRMS data file, both for positive- and negative-mode MS, and based on comparison with the HMDB and the LipidMaps database. Welch’st-tests were executed to evaluate statistical significance. Outcomes of Shapiro–Wilk tests and Levene’s tests are given in Table S2.

**Figure 8:**
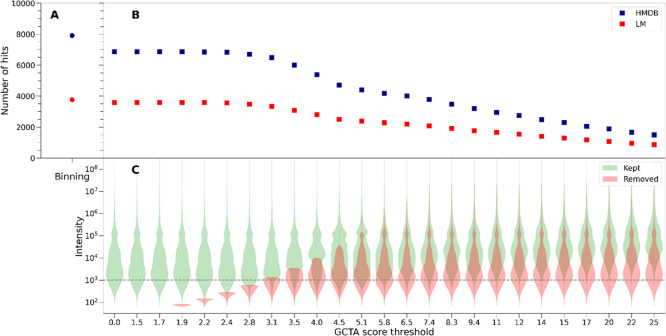
Effect of increasing GCTA score threshold on number of annotations and intensities retained (positive-mode data). B Median number of hits in the HMDB and LipidMaps (LM) database for binning output. For each score threshold, 20 SC HRMS data files were evaluated: in panel **B**, median numbers of hits are given, and in panel **C**, violin plots showing intensities for all data files are given.

SC metabolomics can have a great impact on the elucidation of complex biological phenotypes, but is hampered by the lack of data algorithms suitable for the processing of untargeted HRMS data. Herein, GCTA performance is compared with other clustering algorithms in terms of result similarity, accuracy relative to standard molecular ions and database hits, and likelihood of peak splitting. We demonstrate that GCTA has a similar alignment accuracy as commercially available algorithms when compared to database entries, that GCTA has a lower likelihood of peak splitting than MaldiQuant binning which leads to an increased annotation rate, and that GCTA the scoring function GCTA uses provides a natural means of noise filtering.

## Experimental Method

### Data Generation and Preprocessing

Data was generated using a ThermoFischer Orbitrap Exploris 480 instrument using a scan range of *m*/*z* 50–1100. The ranges *m*/*z* 50–500, 500–1000 and 900–1090 were scanned using full-scan MS. Furthermore, the range 50–980 was also scanned using selected ion monitoring, using a range size of 30. Data was recorded both in positive and negative mode, polarities were processed separately. An automatic gain control target of 120,000 was used, a radiofrequency funnel between 40% and 60%, depending on the *m*/*z* range, was used, and the injection time was selected automatically.

GCTA clustering and alignment were evaluated using standards, Thermo FreeStyle extracted *m*/*z* value and metabolomics database entries as benchmarks. First, 19 quality control (QC) samples containing Pierce FlexMix Calibration Solution (ThermoFischer Scientific) were used for data acquisition: 8 of these FlexMix compounds form a positive molecular ion by proton adduction, and 6 form a negative ion by deprotonation. The constituents and their *m*/*z* values can also be found in Table S8. Furthermore, SC HRMS data from 10 HEK293T cells and 10 HepG2 cells was used. Proprietary Thermo .raw files were converted to .mzML files using the R package mzR.^18^ Savitzky–Golay filtering was used for peak smoothing,^19^ after which the data was centroided and clustered using our own clustering algorithms. The data handling process from smoothing to alignment is also illustrated in Figures 2 and S1–S5. The code used for this is provided in the aforementioned GitHub. For Savitzky–Golay smoothing, only an array with the intensity values to be smoothened needs to be provided, together with a window size (herein: 5) and a polynomial order (herein: 3). Centroiding, clustering and alignment require the *m*/*z* and intensity values to be input as separate vectors, in addition to the parameters discussed in section *The GCTA algorithm*. Smoothing is incorporated as it is typically required for the processing of e.g. TOF-MS data, but given the high resolution of the data used herein, it should not impact the clustering accuracy.

### The GCTA Algorithm

The GCTA algorithm has been programmed in R, but a Python implementation of the function is also provided via the GitHub specified in the *Data and Code Availability Statement*. Two versions of the GCTA algorithm are available as two different functions: *GCTAn* and *GCTAp* for across-scan clustering and across-file alignment, respectively. This also means that *GCTAn* clusters the *m*/*z* values and returns one value per cluster, whereas *GCTAp* forms neigborhoods, containing one *m*/*z* value per file, and returns the same—aligned—*m*/*z* value for all of the different files.

Several parameters need to be defined before application: the user needs to define the expected maximum measurement error *eps* in ppm (cf. Section *Validation*—*General approach*), that defines the maximum distance at which a data point can be considered part of a cluster. This value is dependent on the instrument the user uses: for an Orbitrap MS instrument, we consider a value of 3 ppm for across-scan clustering and a value of 5 ppm for across-sample alignment reasonable. Furthermore, a value for *K* needs to be defined, that is the maximum number of neighboring data points considered; in principle, this should be equal to the number of scans recorded. However, as the data may show missingness, a lower value for *K* may be more appropriate; choosing a value that is higher than the number of scans does not make sense, as it would imply that the same *m*/*z* value can be measured twice in a single scan. The *m*/*z* scale is log-transformed to ensure a constant cluster width; the base for the log-transformation is defined by the user. Furthermore, a score threshold needs to be determined to filter out the noise during the clustering. In the score function provided with the algorithm, the log-transformed intensity is used as well; the base for this log-transformation is defined by the user. Lastly, functions need to be defined or chosen to calculate the neighborhood score and to convert the *m*/*z* values per neighborhood and their intensities to clustered ones.

When working with GCTAn, it requires an array of *m*/*z* values and an array with the corresponding intensities (in addition to the aforementioned input parameter values). The *nn2* R package from RANN^20^ is used to identify the data points within an *eps* radius; in the Python implementation of GCTA, the cKDTree function from the *scipy.spatial* library is used for this purpose. Subsequently, for each *m*/*z* value, a score is calculated as described above, thereby also considering the number of data points in a neighborhood. All neighborhoods with a score below the threshold are removed. Finally, all neighborhoods are looped through, starting with the lowest-scoring neighborhood. To do the actual clustering, *m*/*z* values are assigned cluster IDs: using *GCTAn*, only the median *m*/*z* is given the ID. This is overwritten if this point is part of a next neighborhood. If any of these are part of a next neighborhood it will be overwritten. Weighted sums are calculated for the *m*/*z* and the intensity: weights are either based on the scores previously determined or based on the intensities. In doing so, neighborhoods are converted to clusters, the data volume is reduced and noise is filtered out based on a user-defined minimum for the neighborhood score. *GCTAp*, however, retains more sample information so compound identification can be assessed retroactively: to each of the data points, the weighted mean *m*/*z* per cluster and the original intensity are assigned. Furthermore, sample IDs are retained. For *GCTAp* all neighbors are given the ID, so they are assigned the same *m*/*z* value as well.

### Validation—General Approach

GCTA clustering performance was compared with MaldiQuant binning (further: binning) and DBSCAN. DBSCAN takes two input parameters, namely epsilon and a minimum number of data points per cluster. To ensure a fair comparison, the same epsilon as used for GCTA was used, and a minimum number of 2 was used, as GCTA does not require the user to define a minimum number of data points per cluster. Clustered *m*/*z* values were compared with the theoretical *m*/*z* values of standards, *m*/*z* values extracted by Thermo FreeStyle (across scans) or Sciex MarkerView (across files) software, or HMDB and LipidMaps database hits; in this way, both the current industrial standards and the theoretical values for metabolite *m*/*z* values are used as grounds truths. All validation steps were conducted using both positive- and negative-mode data.

Oftentimes in this work, accuracies are compared using cumulative histograms of errors in ppm; the number of bins and their width *w*_bin_ are determined using the Freedman-Diaconis rule,^21^ given as eq 1. Even though this rule assumes the binned values to be normally distributed, it is generally able to deal well with outliers,^22^ for instance in comparison to Scott’s rule.^23^ In this equation, *q*_75_ and *q*_25_ represent the 75th and 25th quantile of the array *x*, *F* represents a multiplication factor to tune the bin width (should be *F* = 2 theoretically, here *F* = 1 is used) and *n* is the number of elements in *x*.

1 $$w_{bin}=F^{*}\frac{q_{75}\left(x\right)-q_{25}\left(x\right)}{\sqrt[{3}]{n}}$$

To accommodate the large variation in the clustering errors, a logarithmic *x*-axis is used: eq 1 is applied to the log_10_ of the errors, after which the bin edges are determined based on the bin width determined using eq 1, and converted to a linear *x*-axis. Bar heights are normalized per cumulative histogram and plotted in figures like Figure 3. A good performance is indicated by a plot reaching 100% earlier: most of the errors are small and few are large, so more histogram area is located at low errors.

### Validation—Comparison with Standards

First, clustered *m*/*z* values for the constituents of the aforementioned standard mix were compared with the output from Thermo FreeStyle and Sciex MarkerView. Cumulative histogram bar heights were determined using eq 1.

### Validation—Comparison of Across-Scan and Across-File Clustering with Commercially Available Software

GCTA, binning and DBSCAN accuracies for across-scan clustering and across-file alignment were compared using Thermo- and MarkerView-extracted *m*/*z* values as references, respectively, in both positive and negative mode. SC HRMS data files were used for this part of the study. GCTA and binning results are interpolated using the Python *scipy.interpolate* library so a difference curve can be computed. Error distributions are plotted in cumulative histograms using eq 1.

### Validation—Occurrence of Peak Splitting in GCTA, Binning and DBSCAN Clustering and its Effect on Annotation Rate

Peak splitting in across-scan clustering was investigated by comparing how many clustered *m*/*z* values per SC HRMS data file are assigned to a single Thermo-extracted *m*/*z* value. Subsequently, the occurrences of clustered *m*/*z* values are counted per unique *m*/*z* value, and divided by the number of unique Thermo-extracted *m*/*z* values per data file. Assignment of a single clustered *m*/*z* indicates the peak is left intact, higher numbers of peaks are indicative of peak splitting. Statistical (α = 0.05) tests for normality, equal variance and significant difference between two populations were executed using the respective functions from the Python *scipy.stats* library. Shapiro–Wilk tests were executed to assess normality, and depending on the outcome of this test, Bartlett’s or Levene’s tests were executed to assess equality of variance. Finally, Welch’s *t*-tests or independent *t*-tests were executed to determine whether the means were different. The outcomes of these tests are also given in the referenced tables in the Supporting Information. Subsequently, the Thermo-extracted *m*/*z* values that were considered split by either clustering algorithm were compared with both the HMDB and LipidMaps database; the resulting hits are considered annotations missed due to peak splitting.

### Validation—Comparison of Across-File Clustering with HMDB and LipidMaps Database Hits

MS data files for HEK and HepG2 cells (positive and negative mode) were used. Results from GCTA, binning, DBSCAN and MarkerView across-file clustering were compared against the HMDB^24^ and the LipidMaps^25^ database. H^+^, NH_4_^+^, Na^+^ and COOH^–^ adducts and proton losses were considered, ^13^C peaks were not considered. Results were output as molecular formulas and compound names: elemental compositions and *m*/*z* errors were extracted using Python code, among others making use of the Python *re* library.

### Assessment of Noise Filtering and its Effect on Annotation Rate

The effect of the score threshold on filtering was assessed by logarithmically increasing the threshold below which neighborhoods are considered noise; the intensities of the neighborhoods that were removed and that were retained were compared, and the number of hits was monitored as a function of the score threshold.

## Results

First, accuracy was determined for across-scan clustering and across-file alignment of *m*/*z* values corresponding to standards with known *m*/*z* values. The results of this are shown in Figures 3 and S6, respectively. In both the positive- and negative-mode results, DBSCAN seems to perform slightly worse than GCTA and binning in across-scan clustering, whereas GCTA and binning have very similar performances, evident by the large degree of overlap in the curves. The same is observed in Figure S6: DBSCAN performance is less than that of GCTA and binning, although in absolute sense, the errors obtained for all clustering algorithms are within the expected range for an Orbitrap mass analyzer.

Subsequently, the performances of GCTA, binning and DBSCAN are compared against the *m*/*z* values extracted by Thermo FreeStyle software for HEK and HepG2 cellular HRMS data files. The results of this are shown in Figures 4 and S7, considering all errors and only those lower than 5 ppm, respectively. Furthermore, in Table S1, an overview is given of the average percentages of clustering errors below the accuracy threshold of 5 ppm. In Figure 4, it is visible that GCTA and binning again have a very similar performance, and that DBSCAN performance is much less. As shown by the difference curves in Figure 4C,D, GCTA performs slightly better than binning, but the difference is very small. When only errors below 5 ppm are considered (cf. Figure S7), DBSCAN performs slightly better than GCTA and binning, evident by it reaching higher normalized occurrences for lower error values. However, as is apparent from Table S1, significantly less clusters found by DBSCAN meet the accuracy threshold, as opposed to GCTA and binning.

Furthermore, across-file alignment of cellular data files was compared against MarkerView alignment of these data files. Alignment errors between GCTA, binning and DBSCAN across-file alignment and MarkerView alignment are given in Figure 5; only errors below 5 ppm are considered. Here, it is clearly visible that DBSCAN performs better (gives results closer to that of MarkerView) than GCTA and binning, and that GCTA performs slightly better than binning.

To evaluate alignment accuracy independent of any benchmark approach, metabolomics databases HMDB and LipidMaps were screened for the *m*/*z* values resulting from across-file alignment results using GCTA, binning, DBSCAN and MarkerView for cellular data; the results are shown in Figure 6 for the HMDB and Figure S13 for the LipidMaps database. Aligned *m*/*z* values were compared with the theoretical *m*/*z* values for the database hits. As multiple metabolites may have the same elemental composition and clustered *m*/*z* values may give multiple database hits within 5 ppm, each clustered *m*/*z* value was compared to only the database hit closest in mass. In Figure 6, it is visible that GCTA and DBSCAN have a very similar performance in both positive and negative mode, and that all four algorithms generally perform similarly. This is also observed for the clustering errors with respect to the LipidMaps database hits.

GCTA was designed to reduce the chance of peak splitting as compared to binning, which is one of most used clustering methods in the field. The likeliness of peak splitting into 1 (no splitting), 2, 3, ... peaks (cf. Section *Validation—Occurrence of peak splitting in GCTA, binning and DBSCAN clustering*) is given in Figure S8 for GCTA vs binning, and in Figure S9 for GCTA vs DBSCAN. Furthermore, in Tables S2 and S3, the outcomes of the statistical tests used to assess the underlying assumptions are given. Figure S8 shows that it is significantly more likely that a peak is kept intact in GCTA, whereas it is more likely to be split in binning in positive mode. In negative mode, however, this difference in likelihood of peak splitting is not found. Furthermore, Figure S9 shows that peak splitting is less likely to occur in DBSCAN than it is in GCTA, both in positive and in negative mode. Subsequently, the effect of peak splitting by either algorithm on the annotation rate per SC HRMS data file was investigated. The results of this are given in Figure 7, outcomes of the underlying statistical tests are given in Table S4. In this figure, it is visible that for both positive- and negative-mode data, the number of missed annotations is lower for GCTA than it is for binning, both when comparing with the HMDB and with the LipidMaps database. The numbers of missed annotations normalized with respect to the number of peaks in the GCTAn output are compared in Figure S10, the output of the corresponding statistical tests is given in Table S5. Also in this case, GCTA performs significantly better than binning for both polarities and for both databases. In Figure 7, multiple annotations can be considered per *m*/*z* value; Figure S11 shows the same data, however only considering one annotation per input *m*/*z* value. The results of the corresponding statistical tests are given in Table S6. Also here, GCTA performs significantly better than MaldiQuant binning for both databases and both polarities. Lastly, when the numbers of annotations are corrected for both the numbers of peaks in the GCTAn output, and a maximum of one annotation per input *m*/*z* value is used, GCTA still outperforms binning; this is shown in Figure S12 and Table S7.

Lastly, the noise filtering functionality of GCTA that results from the score threshold was evaluated. To this end, as described in section *Assessment of noise filtering and its effect on annotation rate*, the effect of different score thresholds on both the number of hits and the intensities that were removed and retained was evaluated. The result of this is given in Figure 8 for positive-mode data; the result for negative-mode data is given as Figure S14 in the Supporting Information. In Figure 8B, it can be seen that the number of hits decreases for increasing score threshold: first slowly, but later more rapidly. Furthermore, in Figure 8C, it can be seen that the intensities that are removed tend to have higher and higher values for increasing score threshold. In addition to that, from the overlap between the violin plots it can be seen that for almost all score thresholds, data points with lower intensities are removed, whereas data points with higher intensities are retained.

## Conclusions and Discussion

HRMS is the technique of choice in SC metabolomics studies but HRMS data processing is hampered by the lack of *m*/*z* clustering and alignment techniques suitable for untargeted SC HRMS data. As a consequence, peaks that correspond to the same compound but that have slightly different *m*/*z* values due to scan-to-scan or sample-to-sample variability, can be considered different compounds, which makes feature identification impossible. Existing clustering algorithms are likely to either split or aggregate peaks, and alignment techniques typically require a reference spectrum which is not available in SC metabolomics studies. Peak splitting and aggregation lead to *m*/*z* inaccuracies in peak identification and suggest that more or less (respectively) peaks (compounds) are present than measured. To address this, the focus is on *m*/*z* data clustering and alignment. The GCTA algorithm does both, in a very similar way: clustering across scans, and reference-free alignment across files. Furthermore, proper choice of the score threshold used for neighborhood ranking also enables the user to filter out the noise. The output of GCTA, however, mostly depends on the choice of the values for *K* and *eps*; initial estimates for these values can be made based on the experimental setup (cf. Section *The GCTA algorithm*), further optimization of these values can be done visually by comparing the raw spectra with the clustering results and checking for (missed) peaks. Therefore, after application of GCTA, a data matrix can be generated that lists the intensities of each *m*/*z* value in each sample, ready for further data analysis.

Comparison of Figures 4 and S7 shows that DBSCAN is slightly more accurate than GCTA when it comes to across-scan clustering, but only when the largest errors have been removed from the clustering output; this is true for both positive- and negative mode data. This is also reflected by Figure 3 and Table S1. This is potentially due to peak aggregation in DBSCAN: peak aggregation is prevented in GCTA by proper choice of the value of *K*, whereas DBSCAN may consider multiple closely spaced high-density regions to be one peak. This leads to a large clustering error. Furthermore, in GCTA, neighborhoods of which the score does not exceed the score threshold are removed, which prevents the inclusion of slightly denser, noisier regions of the *m*/*z* range to be included as peaks. Figure 5, however, shows a much larger difference in accuracy of across-file clustered *m*/*z* values between GCTA and binning on one hand, and DBSCAN on the other. This shows that DBSCAN does a better job at aligning *m*/*z* values than GCTA and binning do. However, GCTA is primarily a clustering algorithm. Furthermore, the function that GCTA uses to align across-scan clustered *m*/*z* values can also be tuned and optimized by the user, so the final result becomes more similar to the binning performed in MarkerView. It should be noted, however, that when the alignment results are compared with the theoretical values from metabolomics databases (cf. Figures 6 and S13), GCTA and DBSCAN perform very similarly. Therefore, a likely explanation for the results seen in Figure 5 is that DBSCAN may be closer to sorting-based binning as is done in MarkerView, but not necessarily closer to the true *m*/*z* value.

Figure S8 shows that peak splitting is more likely to occur using binning than using GCTA in the clustering of positive-mode data. This difference is not observed in negative-mode data; this is probably due to the fact that less peaks were detected in negative mode, so the likelihood of peaks being present at the edges of bins decreases and the extent of splitting is reduced. As a consequence, the advantage of GCTA over binning in preventing peak splitting is balanced out. DBSCAN, however, is shown to be less likely to split peaks than GCTA (cf. Figure S6). By design, DBSCAN is more likely than GCTA to identify a cluster of data points as a peak; therefore, it is more likely to identify more peaks, which leads to a lower peak splitting frequency. Furthermore, as shown in Figure 7, this lower likelihood of peak splitting is also translated to less missed annotations when using GCTA vs when using binning, which demonstrates the suitability of GCTA for SC metabolomics data.

Figures 6 and S7 show that the across-file alignment accuracies of the algorithms considered herein have a similar performance, with binning and MarkerView performing slightly better than GCTA and DBSCAN, that in turn perform very similarly. This trend, however, is not observed for negative-mode data. Therefore, no strong preference can be supported for any of the clustering algorithms considered herein based on the data presented.

Lastly, the effect of the choice of the score threshold on filtering noise vs filtering annotations was investigated. In this figure, it can be seen that a score threshold of approximately 3 filters out mostly noise (judging from the intensities removed, Figure 8C) and hardly affects the number of annotations (Figure 8B). Furthermore, given the intensities of the data points filtered out, most likely the data points that were considered hits were no hits, but rather noisy data points that happened to have an *m*/*z* value within 5 ppm of a database entry. Furthermore, the use of a score threshold gives the user a large degree of freedom in which criteria they select to filter noise. The current version of the score function gives lower values to neighborhoods with fewer members or lower intensities, meaning that low-intensity metabolites (that are measured multiple times) can be retained, whereas high-intensity noise (that is only measured in a few scans) is removed. This integration of data clustering and noise filtering makes GCTA uniquely suitable for the clustering of noise HRMS data, such as in SC metabolomics.

GCTA runs very efficiently: loading and preprocessing a raw data file takes about a second on a normal PC, running GCTAn on the preprocessed data takes around 3 s per file. Running GCTAp takes a similar amount of time, although this may become longer when the number of data files considered increases. The working memory of the user’s PC can be an issue here, although this issue can be alleviated by using a higher score threshold in GCTAn, or by skipping the alignment of the data altogether and working with the database hits the GCTAn output gives.

All in all, it has been shown that GCTA is a suitable algorithm for the clustering and alignment of untargeted HRMS data: GCTA clustering and alignment individually result in highly accurate results with respect to commercially available algorithms. Furthermore, it is less likely to split peaks than binning, and GCTA’s clustering errors with respect to metabolomics database hits are comparable to those of DBSCAN, MarkerView and binning. Lastly, the inclusion of intensities in *m*/*z* value clustering opens the way to more reliable quantitation: currently, mostly only *m*/*z* values are used in clustering, but by also considering the intensity values, the user can choose or define a function to also determine clustered intensity values, which can improve the quantification quality. GCTA enables the reference-free clustering and alignment of HRMS *m*/*z* values in untargeted SC metabolomics experiments, and is therefore essential to the maturation of this developing and impactful field.

## Supplementary Material



## Notes

Pseudocode of the algorithm, examples of data preprocessing, clustering and alignment, comparisons of clustering accuracies and peak splitting likelihoods, tables listing average percentages of clustering accuracies below the accuracy threshold and standard mix constituents (PDF)

## ⊥

Deceased.

## Notes

GCTA consists of two functions, written in R, the code of both functions is available via GitHub (https://github.com/DirkWevers/GCTA/releases/tag/v1.0). Furthermore, in the same GitHub, a Python implementation of the GCTA algorithm is provided, together with a short manual on how to run this code in a Python environment. Lastly, parts of the code essential to data processing (e.g., centroiding, smoothing, error calculations) have been made available. The raw HRMS data used for this publication and the output from MarkerView and FreeStyle are available via Zenodo (ID: 10.5281/zenodo.17018308).
