# Supplementary material for: An m/z- and Intensity-Based HRMS Clustering Algorithm Targeted toward Single-Cell Metabolomics
Source: J Am Soc Mass Spectrom. 2026 Jul 10;37(8):1942–51. doi: 10.1021/jasms.6c00150 (PMC13450477; doi:10.1021/jasms.6c00150)
Supplement: Supplementary file 1 [file js6c00150_si_001.pdf]

Supplementary information - An *m/z*- and intensity-based HRMS clustering algorithm targeted towards single-cell metabolomics

Dirk Wevers[1‡], Laura Koldenhof[1,2‡], Laura Castaneda[1], Anouschka van der Zeeuw[3], Hellen Fass[1†], Thomas Hankemeier[1], Charlie Clark[1], Ahmed Ali[1*]

[1] Metabolomics and Analytics Centre, Leiden Academic Centre for Drug Research, Wassenaarseweg 76, 2333 AL Leiden, The Netherlands

[2] Universiteit Utrecht, Heidelberglaan 8, 3584 CS Utrecht, The Netherlands

[3] Hogeschool Leiden, Zernikedreef 11, 2333 CK Leiden, The Netherlands

[‡] These authors are shared first authors

[†] Deceased

Corresponding author: Ahmed Ali, ali@lacdr.leidenuniv.nl

Table of contents **Page**

Code snippet S1: Pseudo-code for *GCTAn* *S-3*

Figure S1: Overview of data clustering and alignment done herein *S-4*

Figure S2: Overview of data pre-treatment, clustering and alignment done herein, shown for a HRMS spectrum recorded for a HEK293T cell ($C_{35}H_{65}O_{10}P+H^+$) *S-5*

Figure S3: Overview of data pre-treatment, clustering and alignment done herein, shown for a HRMS spectrum recorded for a HEK293T cell ($C_{43}H_{76}NO_8P+H^+$) *S-6*

Figure S4: Overview of data pre-treatment, clustering and alignment done herein, shown for a HRMS spectrum recorded for a HEK293T cell ($C_{31}H_{58}NO_{11}P+H^+$) *S-7*

Figure S5: Overview of data pre-treatment, clustering and alignment done herein, shown for a HRMS spectrum recorded for a HEK293T cell ($C_{11}H_{20}O_4+H^+$) *S-8*

Figure S6: Alignment errors for GCTA, binning and DBSCAN for standards in QC files in positive and negative mode *S-9*

Figure S7: Clustering errors for GCTA, binning and DBSCAN relative to Thermo-extracted *m/z* values in cellular MS data files in positive and negative mode. *S-9*

Table S1: Percentages of clustering errors below the threshold of 5 ppm for GCTA, binning and DBSCAN *S-9*

Figure S8: Peak splitting in GCTA *vs.* binning in SCMS data in positive and negative mode *S-10*

Table S2: *p*-values obtained for the statistical tests executed on the data shown in Figure S8. *S-10*

Figure S9: Peak splitting in GCTA *vs.* DBSCAN in SCMS data in positive and negative mode *S-10*

Table S3: *p*-values obtained for the statistical tests executed on the data shown in Figure S9. *S-11*

Table S4: *p*-values obtained for the statistical tests executed on the data shown in Figure 7. *S-11*

Figure S10: Numbers of missed annotations due to peak splitting by GCTA or MaldiQuant binning, corrected for the number of peaks in the GCTAn output. *S-11*

Table S5: *p*-values obtained for the statistical tests executed on the data shown in Figure S10. *S-11*

Figure S11: Numbers of missed annotations due to peak splitting by GCTA or MaldiQuant binning, considering one annotation per input *m/z* value. *S-12*

Table S6: *p*-values obtained for the statistical tests executed on the data shown in Figure S11. *S-12*

Figure S12: Numbers of missed annotations due to peak splitting by GCTA or MaldiQuant binning, corrected for the number of peaks in the GCTAn output and only considering one annotation per input *m/z* value. *S-12*

Table S7: *p*-values obtained for the statistical tests executed on the data shown in Figure S12.	*S-13*
Figure S13: Clustering errors with respect to nearest LipidMaps hits for GCTA, binning and DBSCAN for positive- and negative-mode SCMS data	*S-13*
Figure S14: Numbers of hits and removed and retained intensities for increasing GCTA score threshold, data acquired in negative mode.	*S-13*
Figure S15: Explanation of peak aggregation and splitting in GCTA, binning and DBSCAN	*S-14*
Figure S16: Schematic of the data processing workflow in this work.	*S-14*
Table S8: Components and their *m/z* values per ionization mode in the Pierce™ FlexMix™ Calibration Solution manufactured by ThermoFischer Scientific	*S-15*

Code snippet S1: Pseudo-code for *GCTAn*

```
FUNCTION GCTAn(inmz, inint, eps=5, mzbase=2, intbase=10, mzfun='weighted.mean', intfun='weighted.mean', threshold=2,
weights='score', k=110, info=FALSE):

# Step 1 - Data cleaning
Remove all points where inint <= 0
inmz  ← inmz corresponding to positive inint
inint ← inint > 0

IF info THEN
   PRINT "Calculating fixed radius nearest neighbor"
END IF

# Step 2 - Find indices of K nearest neighbours (disregarding the ppm threshold)
log_mz ← log(inmz, base = mzbase)
fr ← k-nearest neighbors of log_mz (using k = 110)
distances ← fr.nn.dists        # matrix of distances in log space
indices   ← fr.nn.idx          # matrix of neighbor indices

# Step 3 - Determine valid neighbors within eps ppm radius
ppm_threshold ← log(1 + eps / 2e6, base = mzbase)
within_range_mask ← True/False where distances < ppm_threshold

IF info THEN
   PRINT maximum(rowSums(within_range_mask))  # max neighbors per point
END IF

# Step 4 - Remove out-of-range neighbors
Set indices where within_range_mask == FALSE to NA
neighbours ← indices

# Step 5 - Compute scores
logint ← log(inint, base = intbase)
neighbor_counts ← rowSums(within_range_mask, ignoring NA)
score ← neighbor_counts * logint
Set score < threshold to NA

# Step 6 - Initialize output arrays
outmz  ← array of NA, same length as score
outint ← array of NA, same length as score

# Step 7 - Establish processing order
IF info THEN
   PRINT "Establishing order"
END IF
ord ← indices sorted by increasing score, ignoring NA

# Step 8 - Select weighting scheme for m/z calculation
IF weights == 'intensity' THEN
   weights ← logint
ELSE IF weights == 'score' THEN
   weights ← score
END IF

# Step 9 - Clustering loop
IF info THEN
   PRINT "Starting clustering loop"
END IF

FOR each point oid in ord (from lowest to highest score):
   neigh ← neighbours[oid` ]  # indices of neighbors
   outmz[neigh]  ← NA          # clear previous lower-score assignments
   outint[neigh] ← NA
   outmz[oid]  ← weighted mean of inmz[neigh], using weights[neigh]
   outint[oid] ← weighted mean of inint[neigh], using score[neigh]
END FOR
```

```
# Step 10 - Compile final cluster data
valid ← indices where outmz is not NA
cluster.mz ← outmz[valid]
cluster.intensity ← outint[valid]
cluster_table ← table with columns (mz, intensity)

RETURN cluster_table
END FUNCTION

# --- MAIN PROGRAM ---

1. Read CSV file '20241009 Test data GCTA.csv'
2. Extract:
   mz_vals ← column 'm.z'
   I_vals  ← column 'Intensity'
3. Compute clustered output:
   C ← GCTAn(mz_vals, I_vals)   # uses default parameters
4. Save result as Excel file:
   '20241009 GCTA output (simulated data).xlsx'
```

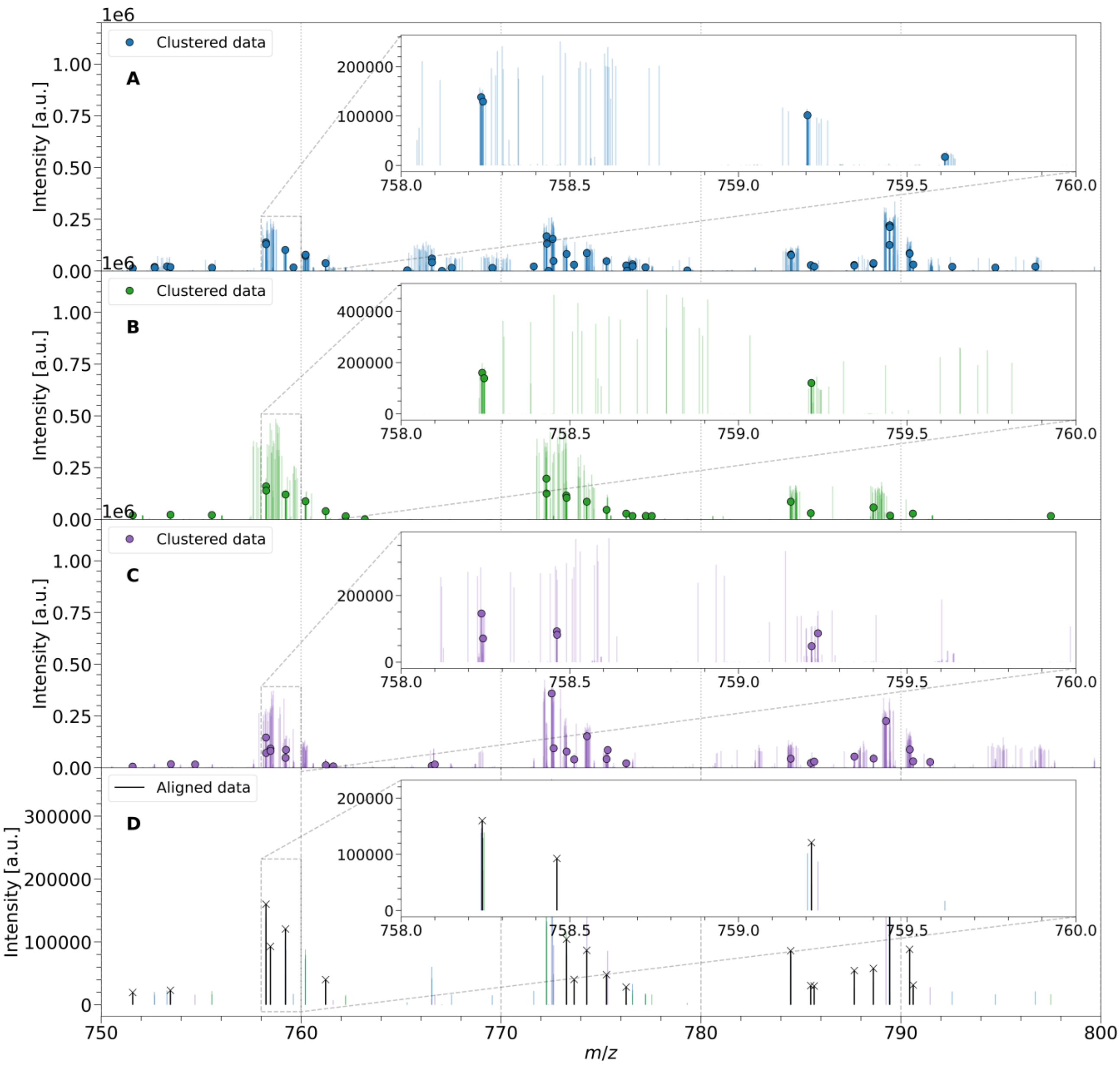

Figure S1: Overview of data clustering and alignment done herein. **A-C** Clustering results for three randomly chosen untargeted SC metabolomics HRMS data files (one file per panel); transparent lines give the data points per scan, clustering results are opaque and are marked with a circle. **D** Alignment results for the clustered data points shown in figure **A-C**; aligned values are marked with a cross.

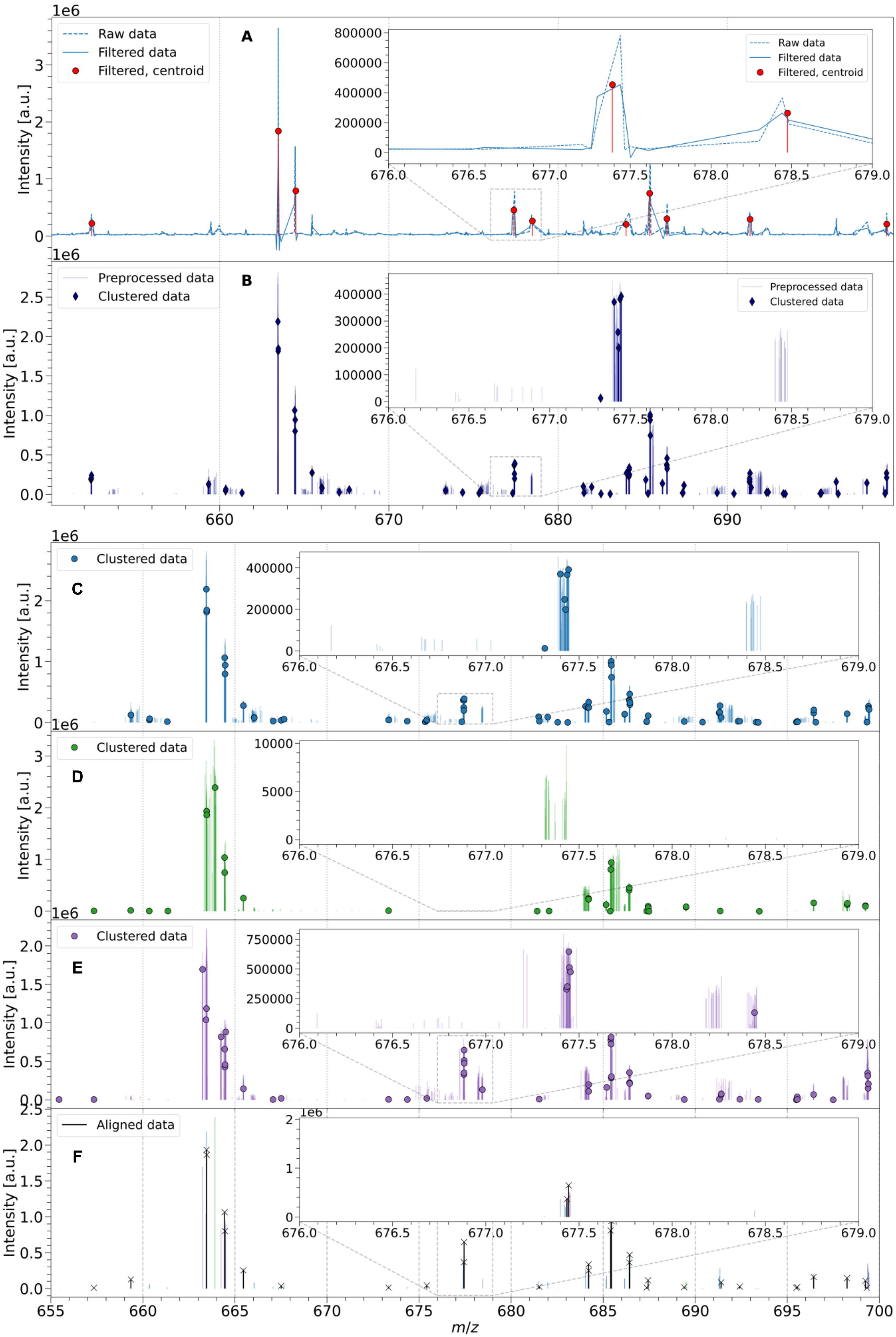

Figure S2: Overview of data pre-treatment, clustering and alignment done herein, shown for a HRMS spectrum recorded for a HEK293T cell. The selected feature is $C_{35}H_{65}O_{10}P+H^{+}$ (m/z_{theor} 677.438, -2.08 ppm), which gives the LipidMaps hit PG 29:2 **A** Effect of Savitzky-Golay filtering and centroiding **B** GCTA clustering results **C-E** Clustering results for three randomly chosen untargeted SC metabolomics HRMS data files; transparent lines give the data points per scan, clustering results are marked with a circle. **F** Alignment results for the clustered data points shown in figure **C-E**; aligned values are marked with a cross.

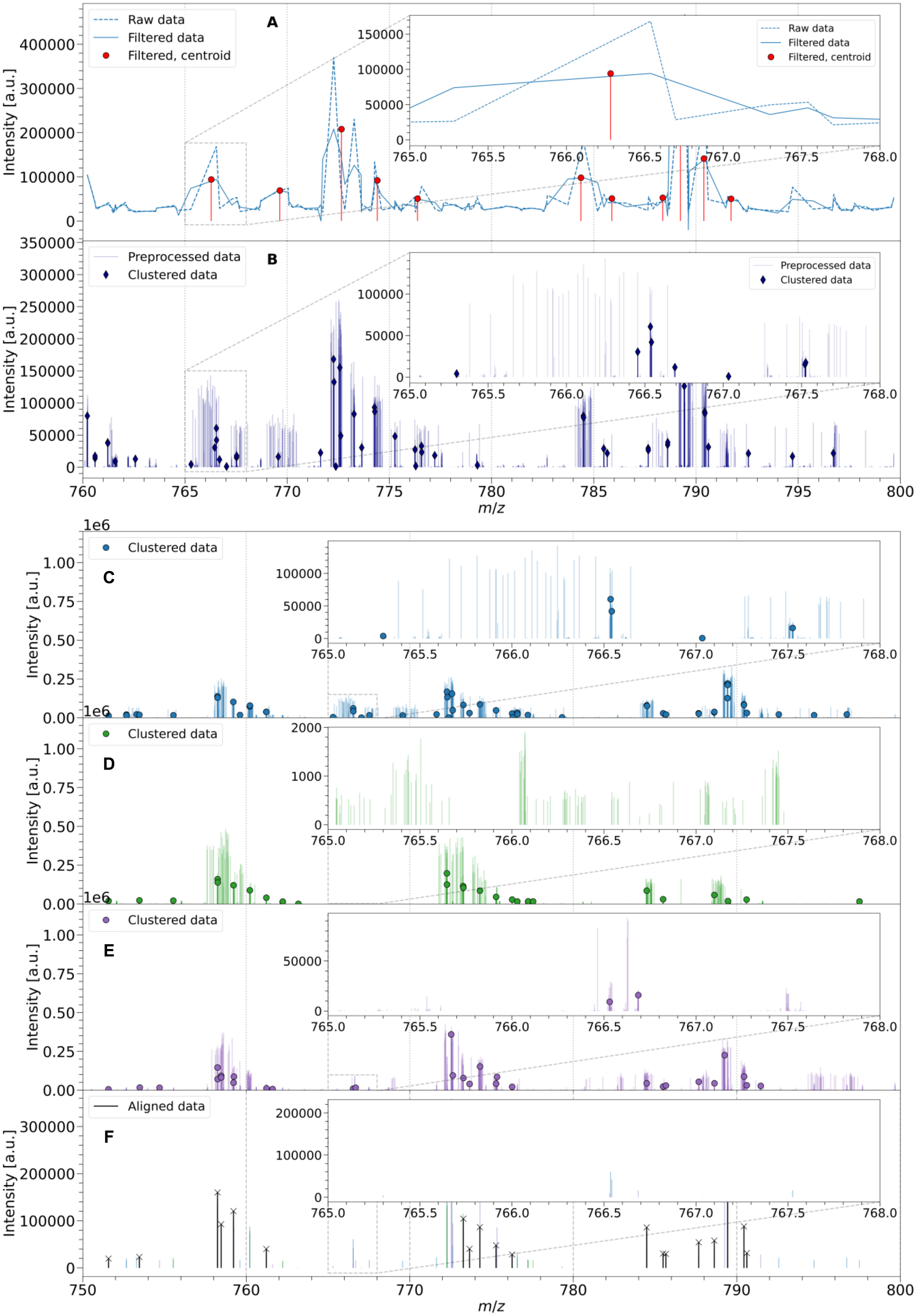

Figure S3: Overview of data pre-treatment, clustering and alignment done herein, shown for a HRMS spectrum recorded for a HEK293T cell. The selected feature is $C_{43}H_{76}NO_8P{+}H^+$ (m/z_{theor} 766.538, -0.04 ppm), which gives the LipidMaps hits PC 35:5, PE 38:5 and PE O-38:6;O **A** Effect of Savitzky-Golay filtering and centroiding **B** GCTA clustering results **C-E** Clustering results for three randomly chosen untargeted SC metabolomics HRMS data files; transparent lines give the data points per scan, clustering results are marked with a circle. **F** Alignment results for the clustered data points shown in figure **C-E**; the clustered values considered here together did not exceed the threshold to be considered for alignment.

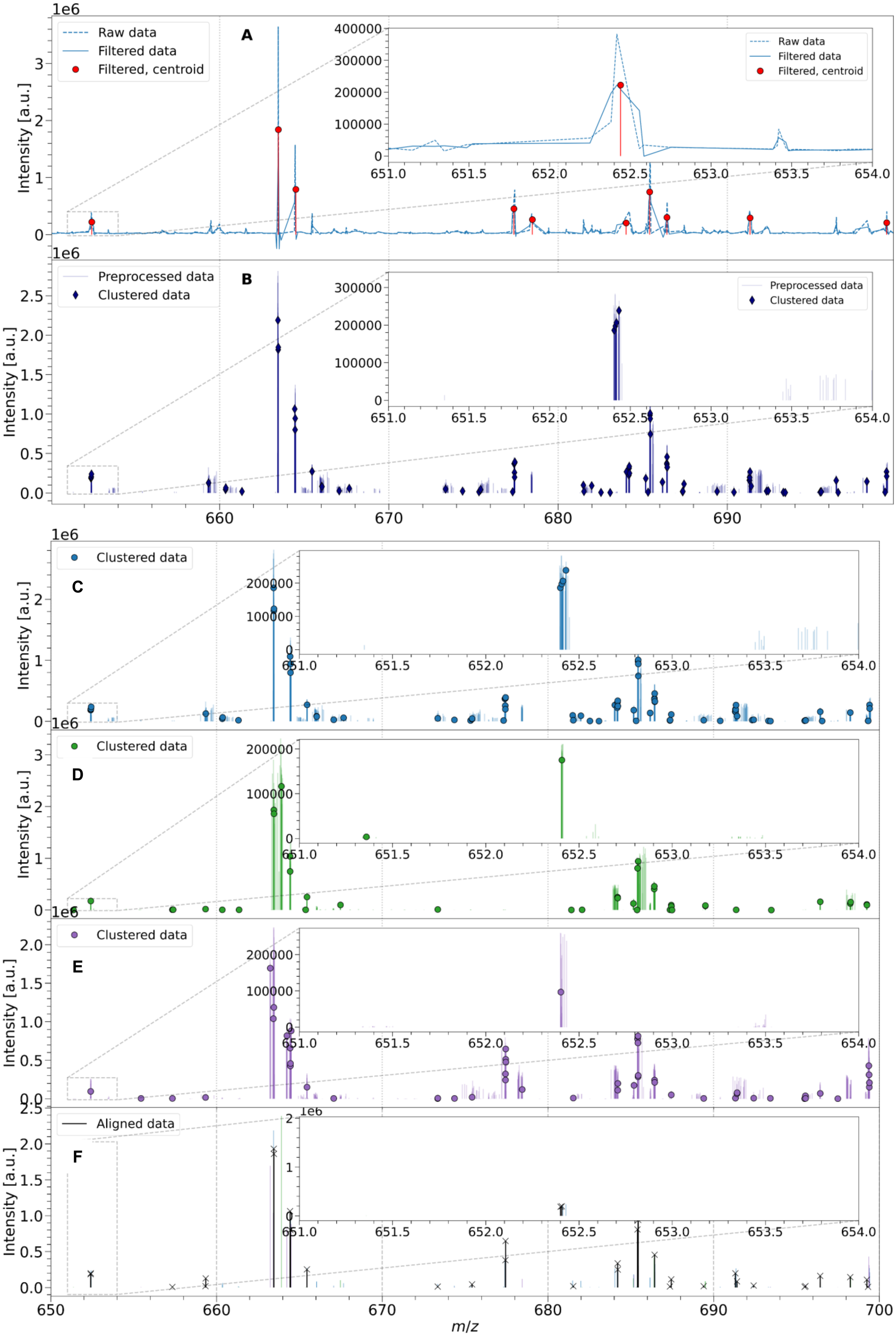

Figure S4: Overview of data pre-treatment, clustering and alignment done herein, shown for a HRMS spectrum recorded for a HEK293T cell. The selected feature is $C_{31}H_{58}NO_{11}P{+}H^{+}$ (0.06 ppm), which gives the LipidMaps hits PC 23:2;O3 and PS 25:1;O) **A** Effect of Savitzky-Golay filtering and centroiding **B** GCTA clustering results **C-E** Clustering results for three randomly chosen untargeted SC metabolomics HRMS data files; transparent lines give the data points per scan, clustering results are marked with a circle. **F** Alignment results for the clustered data points shown in figure **C-E**; aligned values are marked with a cross.

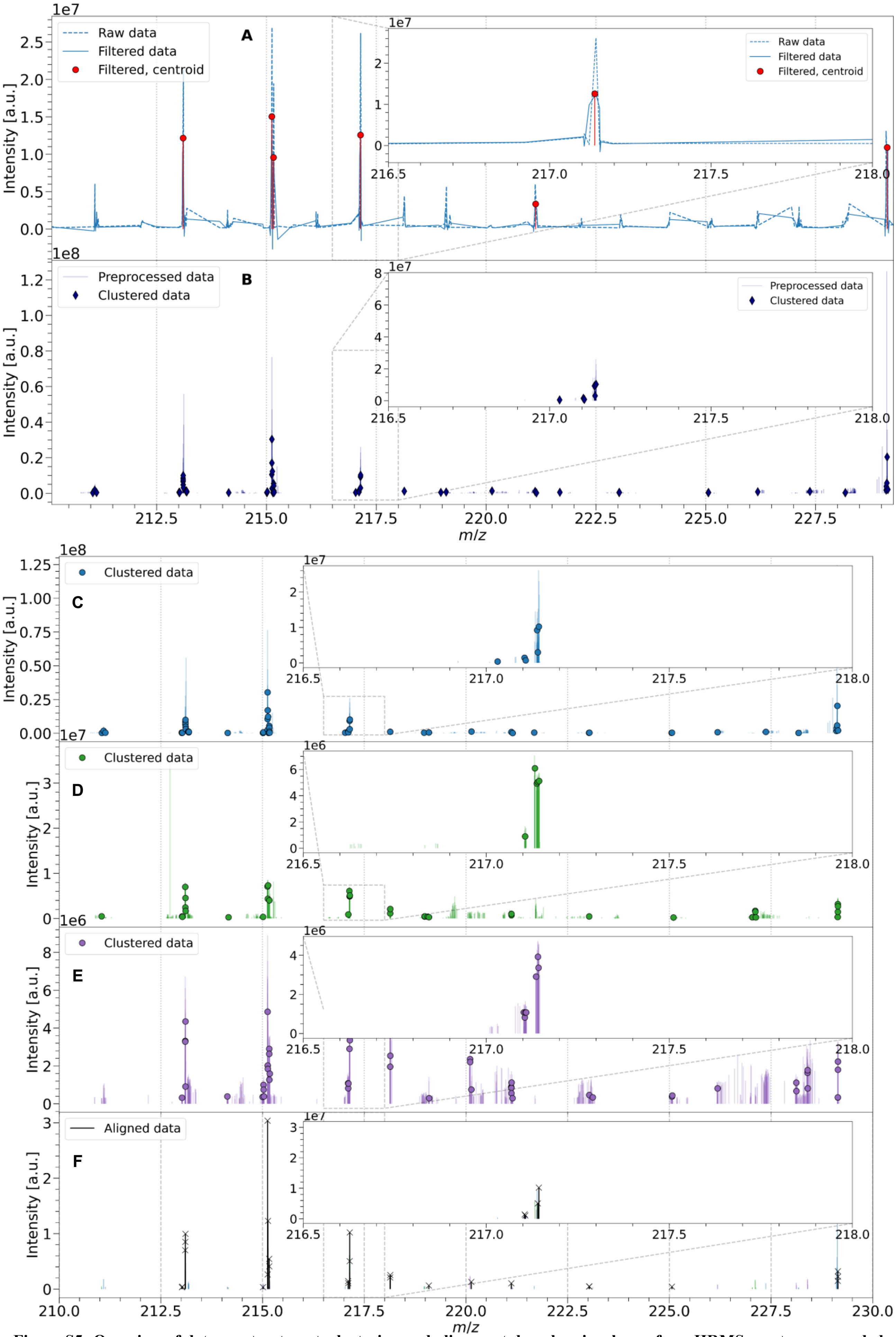

Figure S5: Overview of data pre-treatment, clustering and alignment done herein, shown for a HRMS spectrum recorded for a HEK293T cell. The selected feature is $C_{11}H_{20}O_4$+H^+ (0.61 ppm), which gives the LipidMaps hit SFE 11:1;O2 **A** Effect of Savitzky-Golay filtering and centroiding **B** GCTA clustering results **C-E** Clustering results for three randomly chosen untargeted SC metabolomics HRMS data files; transparent lines give the data points per scan, clustering results are marked with a circle. **F** Alignment results for the clustered data points shown in figure **C-E**; aligned values are marked with a cross.

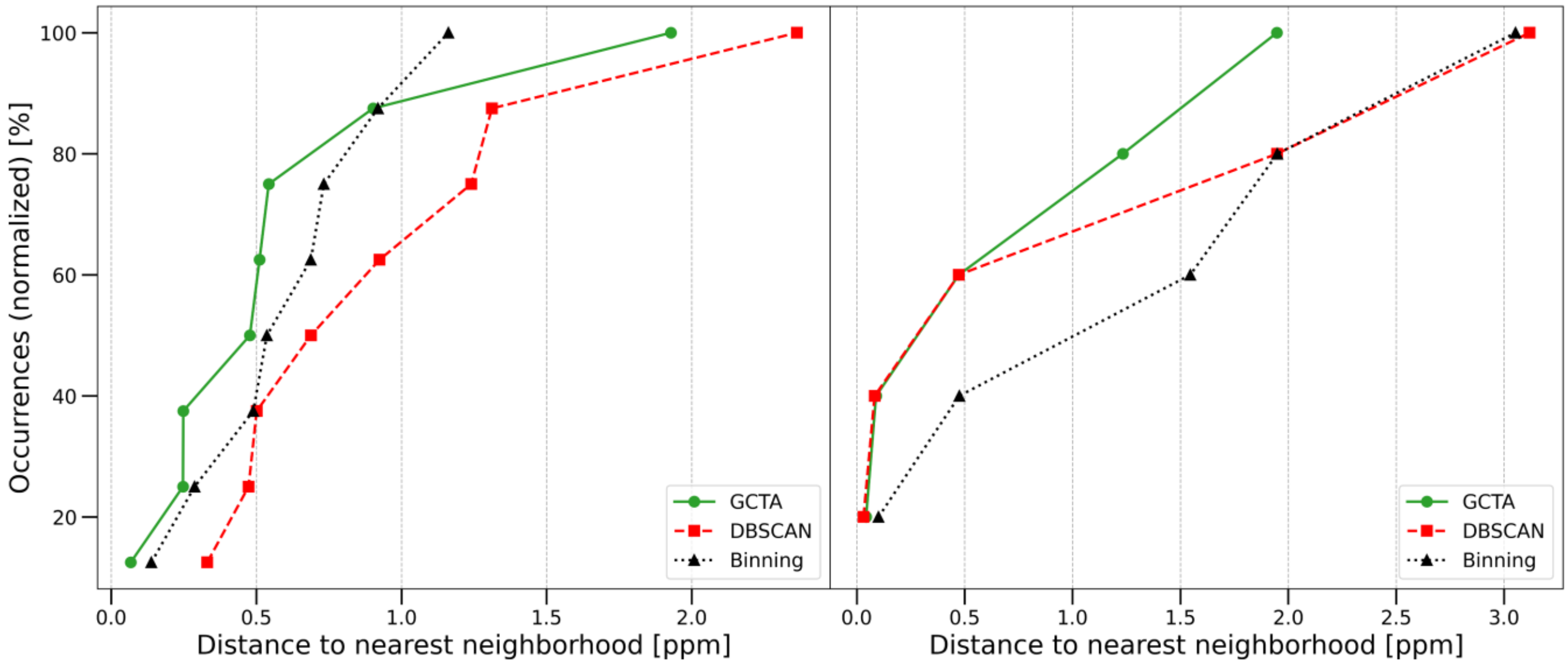

Figure S6: Alignment errors for GCTA, binning and DBSCAN for standards in QC files in positive (left) and negative mode. Data points represent the resulting alignment errors [ppm] after across-scan clustering and across-file alignment, sorted in ascending order.

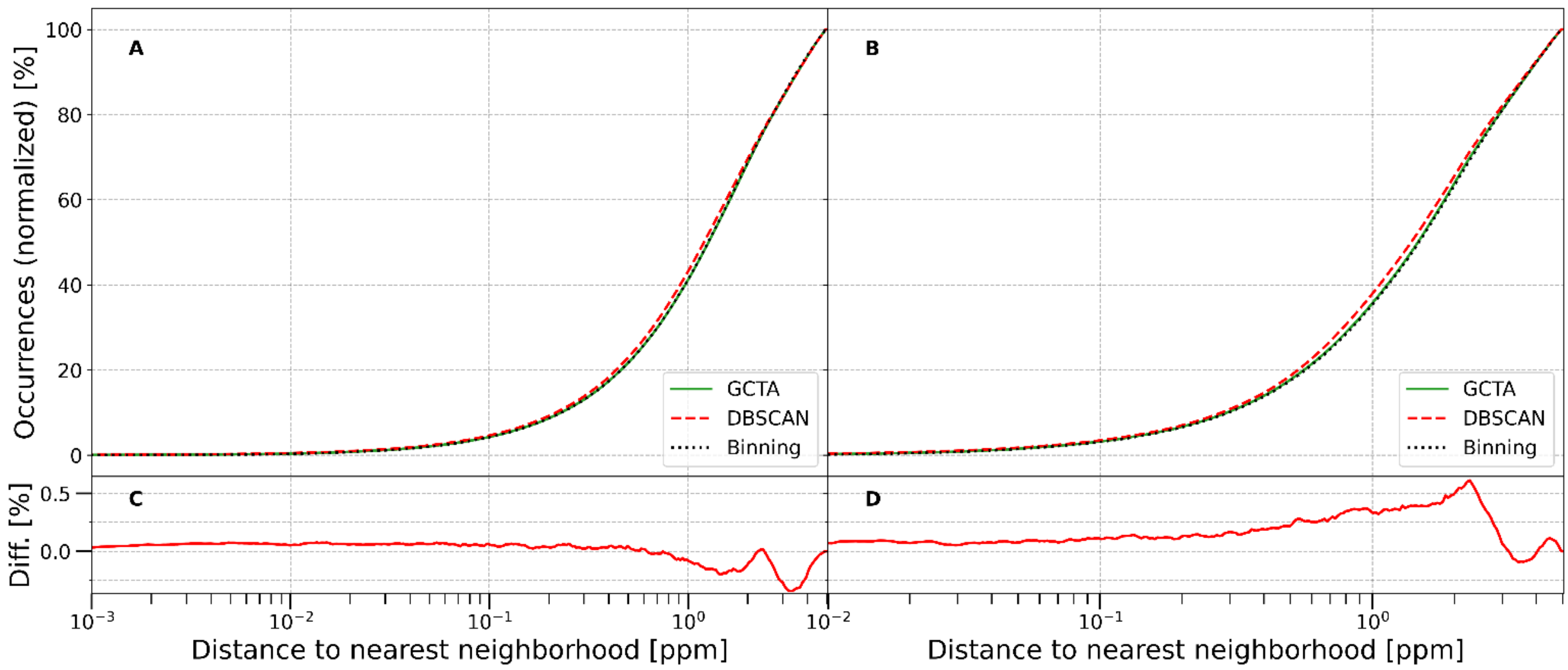

Figure S7: Clustering errors for GCTA, binning and DBSCAN relative to Thermo-extracted *m/z* values in cellular MS data files in (A, C) positive and (B, D) negative mode. Only errors ≤ 5 ppm are considered; percentages of data points below the threshold are given in Table S1. Data points represent normalized heights of cumulative histogram bars generated based on the resulting errors [ppm] after across-scan clustering; bin widths have been calculated using the Freedman-Diaconis rule. Curves reaching higher normalized occurrences earlier indicates lower clustering error and hence better algorithm performance. Figures C and D show the difference GCTA [minus] binning for (C) positive- and (D) negative-mode data. Note the logarithmic x-axis.

Table S1: Percentages of clustering errors below the threshold of 5 ppm for GCTA, binning and DBSCAN. Percentages are calculated relative to Thermo FreeStyle output per polarity. 20 untargeted SC metabolomics HRMS datafiles are used for data processing and calculation of averages and standard deviations. Average values ± standard deviations are given.

	GCTA	Binning	DBSCAN
**Positive**	46.9% ± 3.1%	46.7% ± 3.1%	34.0% ± 2.9%
**Negative**	35.3% ± 3.8%	34.9% ± 3.8%	21.2% ± 3.9%

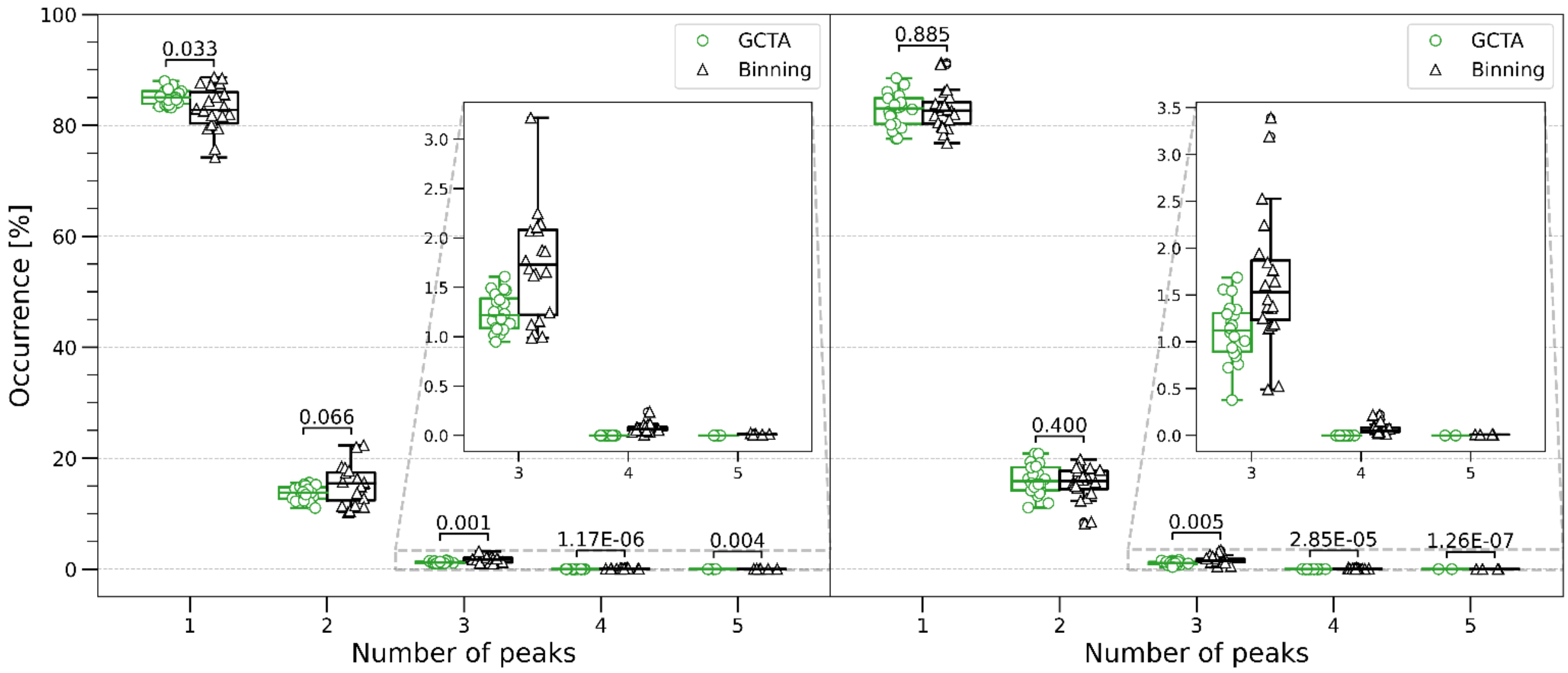

Figure S8: Peak splitting in GCTA *vs.* binning in SCMS data in positive (left) and negative mode. Only peaks with an error ≤ 5 ppm with respect to the nearest Thermo-extracted *m/z* value are considered. Percentages give the numbers of clustered *m/z* values assigned to the same Thermo-extracted *m/z* value, relative to the number of unique Thermo-extracted *m/z* values per clustering algorithm. Higher percentages for $N = 1$ and lower percentages for $N > 1$ indicate a lower likelihood of peak splitting. Shapiro-Wilk tests were executed to assess normality; significant differences were investigated using a two-sided independent t-test or Welch's t-test, depending on the outcome of Levene's test.

Table S2: *p*-values obtained for the statistical tests executed on the data shown in Figure S8. Depending on the outcome of the Shapiro-Wilk (SW) test (α = 0.05), Levene's test or Bartlett's test was executed to assess equality of variance. Depending on the outcome of this test, a Welch's *t*-test or an independent *t*-test was executed to assess whether there is a significant difference in the mean between the two groups.

	SW-test GCTA	SW-test binning	Levene's test	Bartlett's test	Indep. *t*-test	Welch's *t*-test
N=1, +	0,416	0,467	0,001			0,033
N=2, +	0,605	0,352	0,002			0,066
N=3, +	0,579	0,077	0,002			0,001
N=4, +	1,000	0,001		0,00E+00		1,17E-06
N=5, +	1,000	0,230	0,001			0,004
N=1, -	0,909	0,227	0,697		0,885	
N=2, -	0,865	0,046		0,656	0,400	
N=3, -	0,985	0,101	0,031			0,005
N=4, -	1,000	0,001		0,00E+00		2,85E-05
N=5, -	1,000	0,982	0,050		1,26E-07	

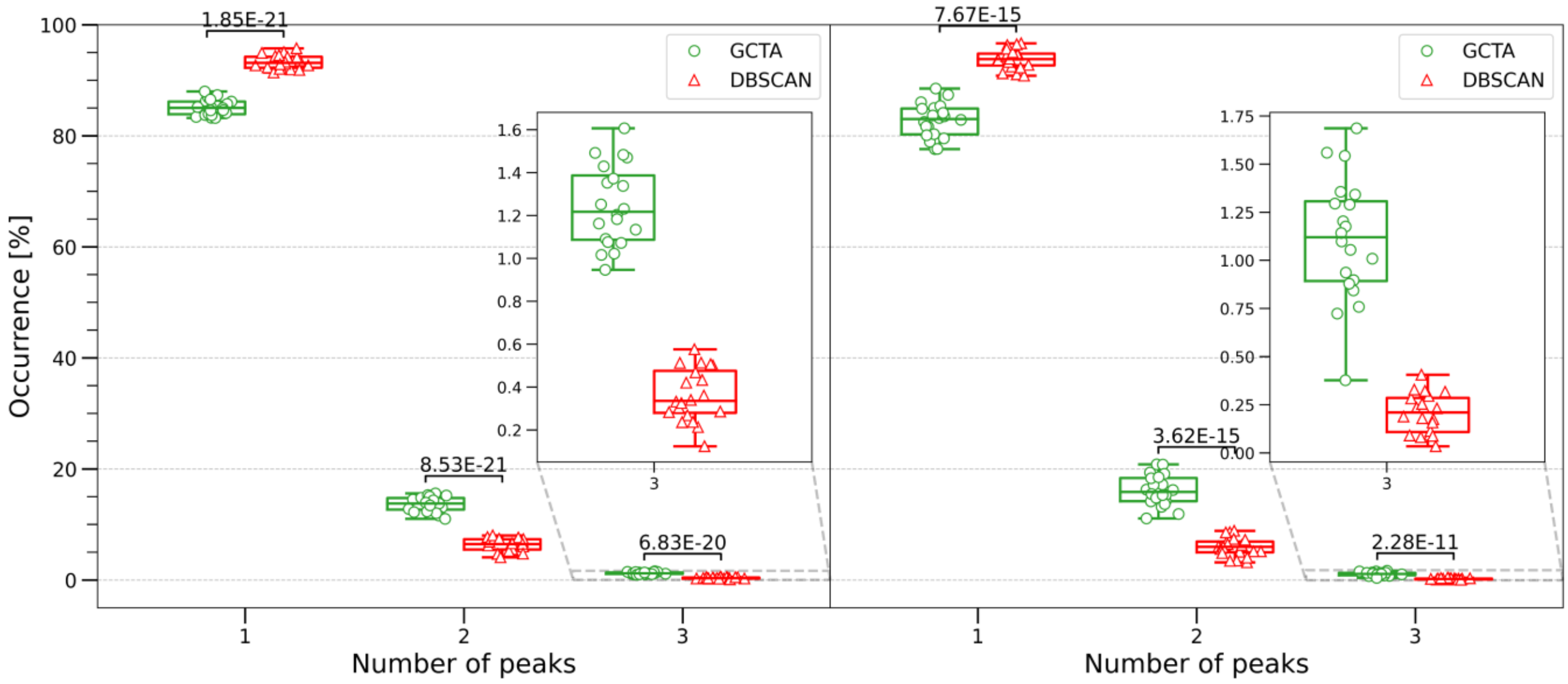

Figure S9: Peak splitting in GCTA *vs.* DBSCAN in SCMS data in positive (left) and negative mode. Only peaks with an error ≤ 5 ppm with respect to the nearest Thermo-extracted *m/z* value are considered. Percentages are calculated based on the number of clustered *m/z* values assigned to the same Thermo-extracted *m/z* value, relative to the number of unique Thermo-extracted *m/z* values per clustering algorithm. Significant differences were investigated using an independent *t*-test or a Welch's *t*-test, depending on the outcome of Levene's test for equal variance.

Table S3: *p*-values obtained for the statistical tests executed on the data shown in Figure S9. Depending on the outcome of the Shapiro-Wilk (SW) test (α = 0.05), Levene's test or Bartlett's test was executed to assess equality of variance. Depending on the outcome of this test, a Welch's *t*-test or an independent *t*-test was executed to assess whether there is a significant difference in the mean between the two groups.

	SW-test GCTA	SW-test DBSCAN	Levene's test	Bartlett's test	Indep. t-test	Welch's t-test
N=1, +	0,416	0,651	0,589		1,85E-21	
N=2, +	0,605	0,554	0,451		8,53E-21	
N=3, +	0,579	0,536	0,056		6,83E-20	
N=1, -	0,909	0,633	0,027			7,67E-15
N=2, -	0,865	0,532	0,037			3,62E-15
N=3, -	0,985	0,645	0,001			2,28E-11

Table S4: *p*-values obtained for the statistical tests executed on the data shown in Figure 7. Depending on the outcome of the Shapiro-Wilk (SW) test ($\alpha = 0.05$), Levene's test or Bartlett's test was executed to assess equality of variance. Depending on the outcome of this test, a Welch's *t*-test or an independent *t*-test was executed to assess whether there is a significant difference in the mean between the two groups.

	SW-test GCTA	SW-test binning	Levene's test	Bartlett's test	Indep. *t*-test	Welch's *t*-test
HMDB, +	0,970	0,493	0,005			8,50E-05
HMDB, -	0,135	0,683	0,011			0,016
LM, +	0,259	0,551	0,001			5,70E-06
LM, -	0,956	0,306	0,017			0,001

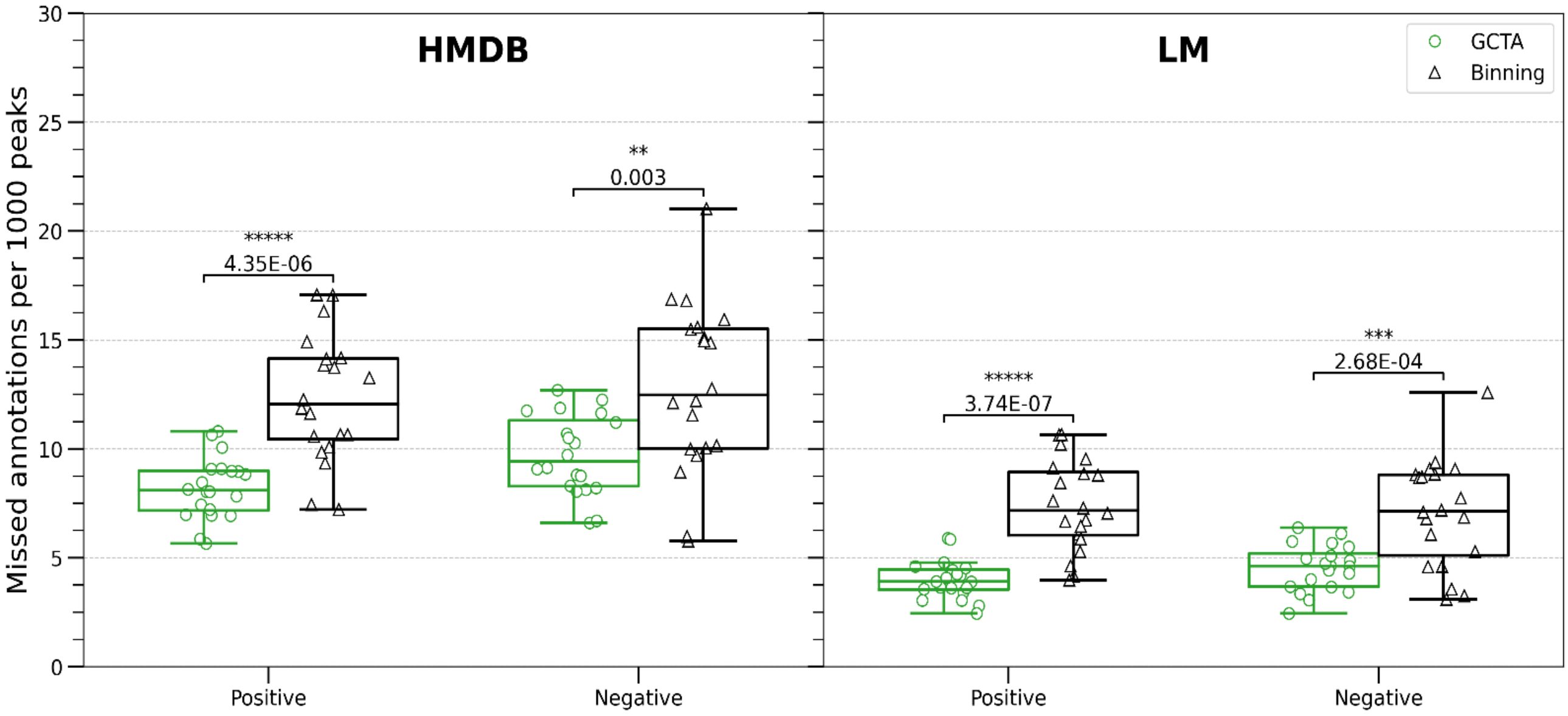

Figure S10: Numbers of missed annotations due to peak splitting by GCTA or MaldiQuant binning, corrected for the number of peaks in the GCTAn output. Numbers of missed annotations are given per SC HRMS data file, both for positive- and negative-mode MS, and based on comparison with the HMDB and the LipidMaps database. Welch's t-tests were executed to evaluate statistical significance, the underlying assumptions were checked using Shapiro-Wilk tests and Bartlett's test, see Table S3.

Table S5: *p*-values obtained for the statistical tests executed on the data shown in Figure S10. Depending on the outcome of the Shapiro-Wilk (SW) test ($\alpha = 0.05$), Levene's test or Bartlett's test was executed to assess equality of variance. Depending on the outcome of this test, a Welch's *t*-test or an independent *t*-test was executed to assess whether there is a significant difference in the mean between the two groups.

	SW-test GCTA	SW-test binning	Levene's test	Bartlett's test	Indep. *t*-test	Welch's *t*-test
HMDB, +	0.767	0.650	0.003			4.35E-06
HMDB, -	0.458	0.648	0.004			3.43E-03
LM, +	0.460	0.537	0.001			3.74E-07
LM, -	0.975	0.327	0.003			2.68E-04

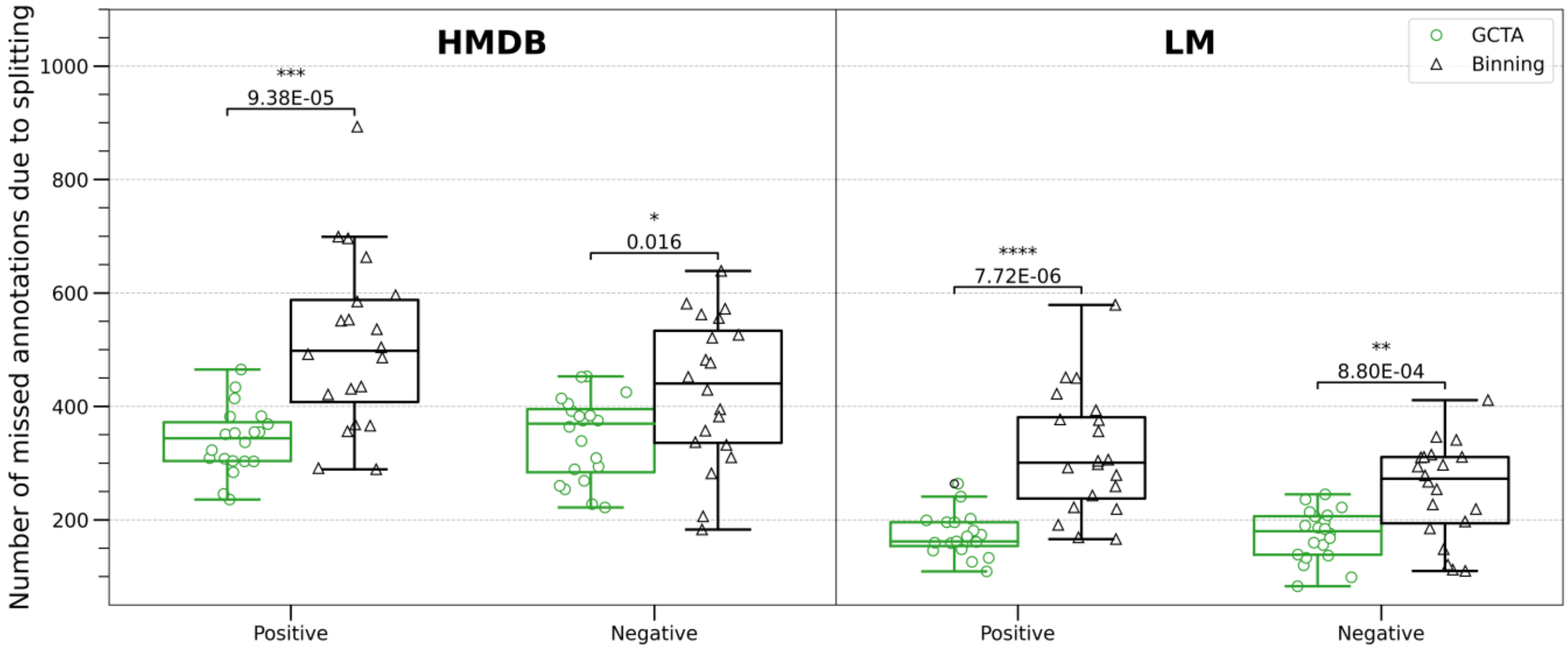

Figure S11: Numbers of missed annotations due to peak splitting by GCTA or MaldiQuant binning, considering one annotation per input *m/z* value. Numbers of missed annotations are given per SC HRMS data file, both for positive- and negative-mode MS, and based on comparison with the HMDB and the LipidMaps database. Welch's *t*-tests were executed to evaluate statistical significance, the underlying assumptions were checked using Shapiro-Wilk tests and Bartlett's test, see Table S6.

Table S6: *p*-values obtained for the statistical tests executed on the data shown in Figure S11. Depending on the outcome of the Shapiro-Wilk (SW) test ($\alpha = 0.05$), Levene's test or Bartlett's test was executed to assess equality of variance. Depending on the outcome of this test, a Welch's *t*-test or an independent *t*-test was executed to assess whether there is a significant difference in the mean between the two groups.

	SW-test GCTA	SW-test binning	Levene's test	Bartlett's test	Indep. *t*-test	Welch's *t*-test
**HMDB, +**	0.865	0.515	0.003			9.38E-05
**HMDB, -**	0.227	0.668	0.016			1.61E-02
**LM, +**	0.280	0.501	0.001			7.72E-06
**LM, -**	0.903	0.314	0.016			8.80E-04

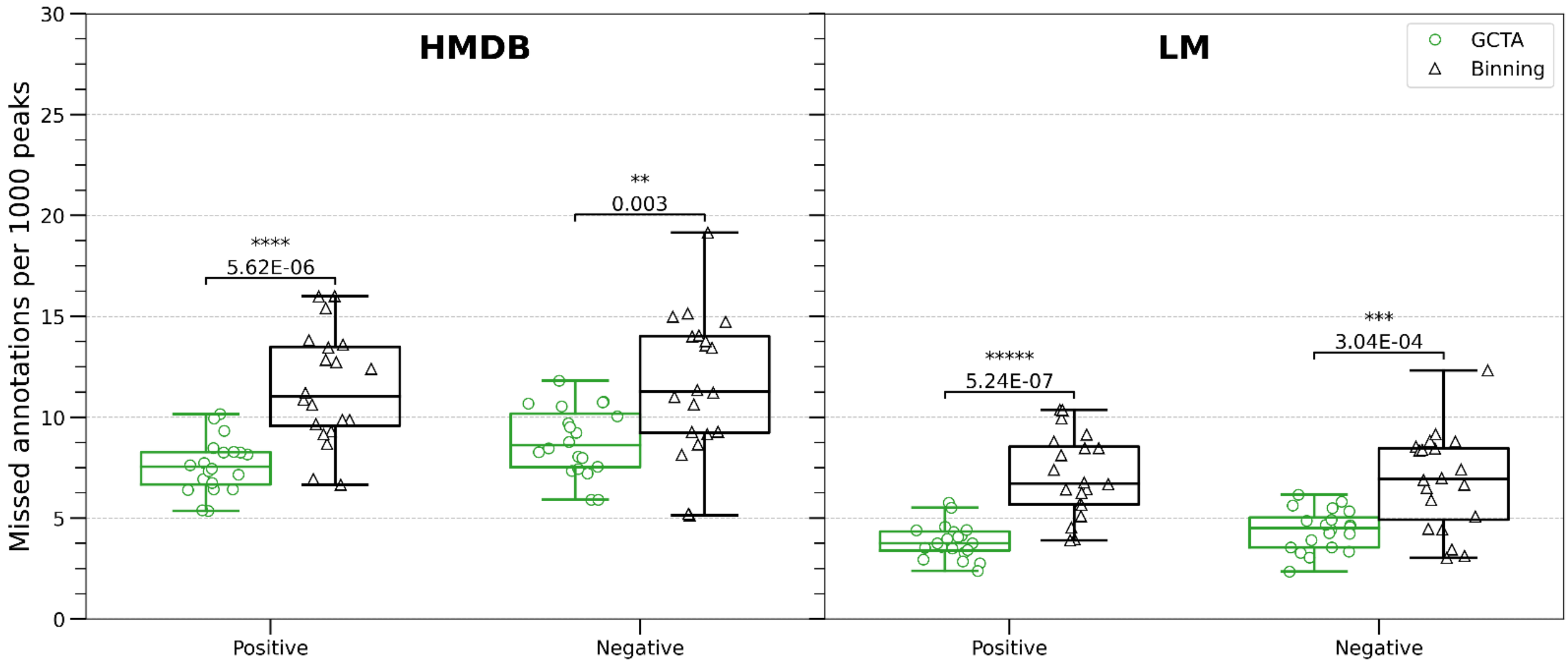

Figure S12: Numbers of missed annotations due to peak splitting by GCTA or MaldiQuant binning, corrected for the number of peaks in the GCTAn output and only considering one annotation per input *m/z* value. Numbers of missed annotations are given per SC HRMS data file, both for positive- and negative-mode MS, and based on comparison with the HMDB and the LipidMaps database. Welch's *t*-tests were executed to evaluate statistical significance, the underlying assumptions were checked using Shapiro-Wilk tests and Bartlett's test, see Table S7.

Table S7: *p*-values obtained for the statistical tests executed on the data shown in Figure S12. Depending on the outcome of the Shapiro-Wilk (SW) test ($\alpha = 0.05$), Levene's test or Bartlett's test was executed to assess equality of variance. Depending on the outcome of this test, a Welch's *t*-test or an independent *t*-test was executed to assess whether there is a significant difference in the mean between the two groups.

	SW-test GCTA	SW-test binning	Levene's test	Bartlett's test	Indep. *t*-test	Welch's *t*-test
HMDB, +	0.706	0.575	0.002			5.62E-06
HMDB, -	0.655	0.586	0.006			3.47E-03
LM, +	0.585	0.475	0.001			5.24E-07
LM, -	0.958	0.364	0.003			3.04E-04

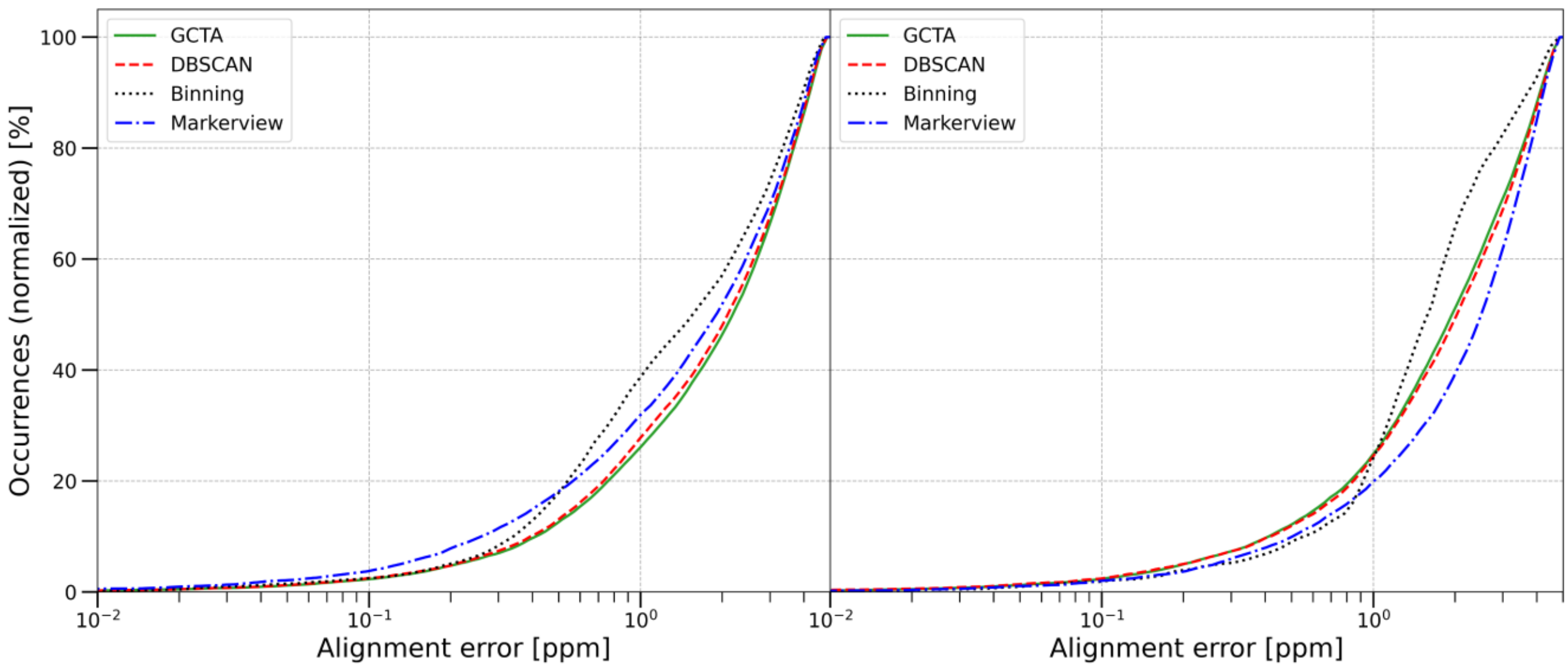

Figure S13: Alignment errors with respect to nearest LipidMaps hits for GCTA, binning and DBSCAN for (left) positive- and (right) negative-mode SCMS data. Only *m/z* values with and error ≤ 5 ppm with respect to the nearest database hit are considered. Data points represent normalized heights of cumulative histogram bars generated based on the resulting errors [ppm] after across-scan clustering; bin widths have been calculated using the Freedman-Diaconis rule.

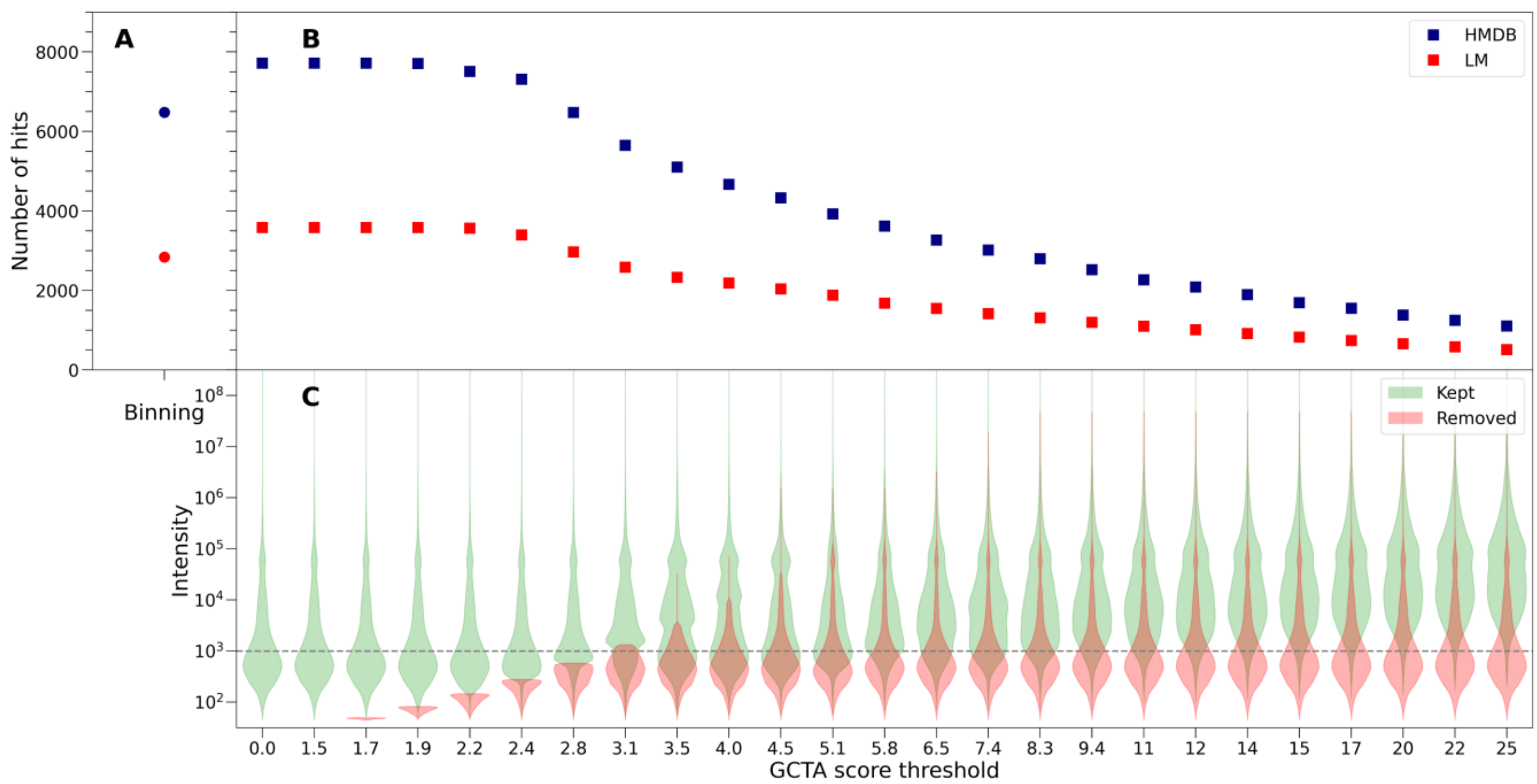

Figure S14: Numbers of hits and removed and retained intensities for increasing GCTA score threshold, data acquired in negative mode. B Median number of hits in the HMDB and LipidMaps (LM) database for binning output. For each score threshold, 20 SC HRMS data files were evaluated: in panel **B**, median numbers of hits are given, and in panel **C**, violin plots showing intensities for all data files are given.

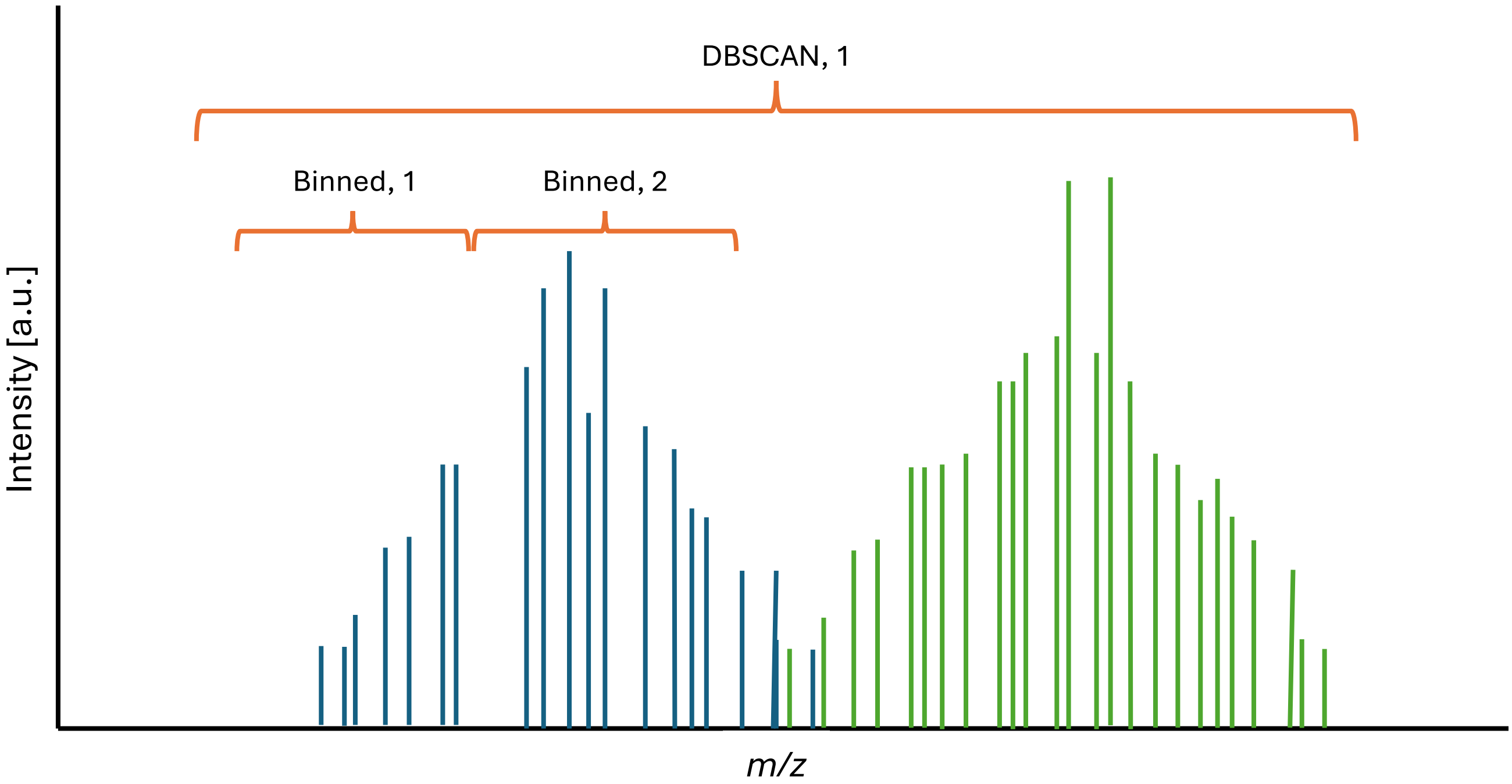

Figure S15: Explanation of peak aggregation and splitting in GCTA, binning and DBSCAN. Due to the overlap between both peaks, DBSCAN is likely to consider them a single peak. Conversely, due to the (randomly) missing values the blue peak and the nature of the binning approach, this peak may be split into two peaks.

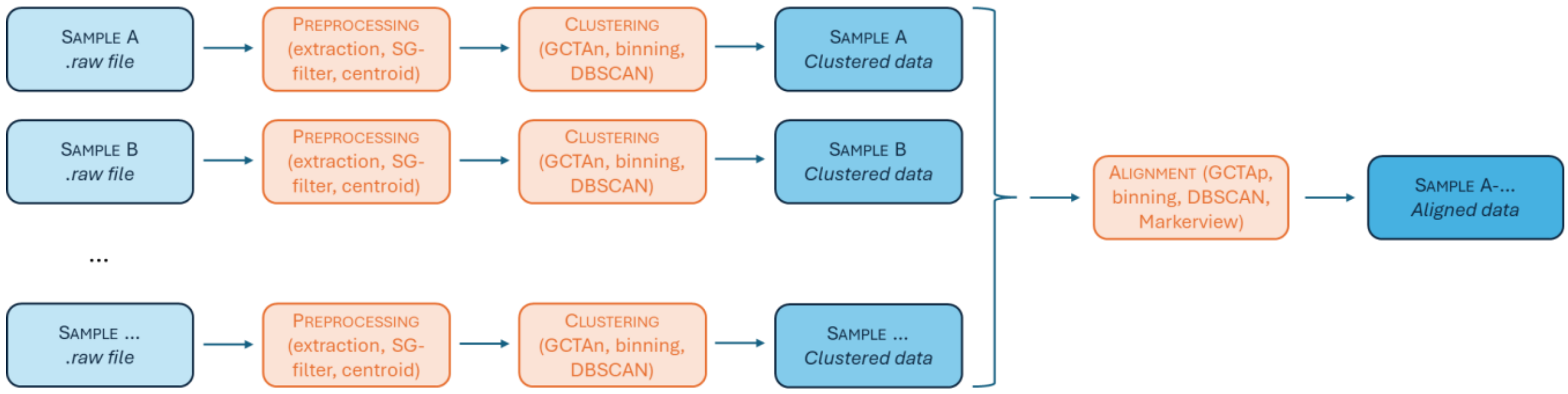

Figure S16: Schematic of the data processing workflow in this work. First, .raw files, each corresponding to a sample, are pre-processed and clustered, resulting in clustered *m/z* and intensity values. Subsequently, the *m/z* values are aligned together, the intensities are conserved.

Table S8: Components and their *m/z* values per ionization mode in the Pierce™ FlexMix™ Calibration Solution manufactured by ThermoFischer Scientific

Components	Positive mode (Accurate Mass m/z)	Negative mode (Accurate Mass m/z)
LC-MS grade acetic acid		59.0128b(-)
Imidazole	69.0447a(+)	
Triethylamine	102.1277a(+)	
Trifluoroacetic acid (TFA)		112.9845b(-)
Tetramethylpiperidine	142.1590a(+)	
Pentafluoropropionic acid		163(-)
Caffeine	195(+)	
Hexamethoxyphosphazene	322.0481a(+)	
Perfluorohexanoic acid		363(-)
MRFA	524(+)	
2,4,6-Tris(heptafluoropropyl)-1,3,5-triazine		601.9779c(-)
Hexakis(2,2-difluoroethoxy)phosphazene	622.0289a(+)	
Hexakis(2,2,3,3-tetrafluoropropoxy) phosphazene	922.0098a(+)	1033.9870d(-)
Ultramark 1621	1022.0034(+)	1233.9742(-)
	1121.9970(+)	1333.9678(-)
	1221.9906(+)	1433.9614(-)
	1321.9842(+)	1533.9550(-)
	1421.9778(+)	1633.9486(-)
	1521.9714(+)	1733.9422(-)
	1621.9650(+)	1833.9358(-)
	1721.9586(+)	1933.9294(-)
	1821.9522(+)	2033.9230(-)
	1921.9458(+)	2133.9166(-)
	2021.9394(+)	
Hexakis(1h,1h,7h-perfluoroheptoxy) phosphazene	2121.9331a(+)	2233.9104d(-)
Hexakis(1h,1h,9h-perfluorononoxy) phosphazene	2721.8948a(+)	2833.8720d(-)
